# Autophagy, ferroptosis, pyroptosis, and necroptosis in tumor immunotherapy

**DOI:** 10.1038/s41392-022-01046-3

**Published:** 2022-06-20

**Authors:** Weitong Gao, Xueying Wang, Yang Zhou, Xueqian Wang, Yan Yu

**Affiliations:** 1 Department of Medical Oncology, Harbin Medical University Cancer Hospital, Harbin, 150081 China; 2 Department of Otolaryngology Head and Neck Surgery, Xiangya Hospital, Central South University, changsha, 410008 China; 3 Department of Head and Neck Surgery, Harbin Medical University Cancer Hospital, Harbin, 150081 China

**Keywords:** Tumour immunology, Drug development

## Abstract

In recent years, immunotherapy represented by immune checkpoint inhibitors (ICIs) has led to unprecedented breakthroughs in cancer treatment. However, the fact that many tumors respond poorly or even not to ICIs, partly caused by the absence of tumor-infiltrating lymphocytes (TILs), significantly limits the application of ICIs. Converting these immune “cold” tumors into “hot” tumors that may respond to ICIs is an unsolved question in cancer immunotherapy. Since it is a general characteristic of cancers to resist apoptosis, induction of non-apoptotic regulated cell death (RCD) is emerging as a new cancer treatment strategy. Recently, several studies have revealed the interaction between non-apoptotic RCD and antitumor immunity. Specifically, autophagy, ferroptosis, pyroptosis, and necroptosis exhibit synergistic antitumor immune responses while possibly exerting inhibitory effects on antitumor immune responses. Thus, targeted therapies (inducers or inhibitors) against autophagy, ferroptosis, pyroptosis, and necroptosis in combination with immunotherapy may exert potent antitumor activity, even in tumors resistant to ICIs. This review summarizes the multilevel relationship between antitumor immunity and non-apoptotic RCD, including autophagy, ferroptosis, pyroptosis, and necroptosis, and the potential targeting application of non-apoptotic RCD to improve the efficacy of immunotherapy in malignancy.

## Background

**Fig. 1:**
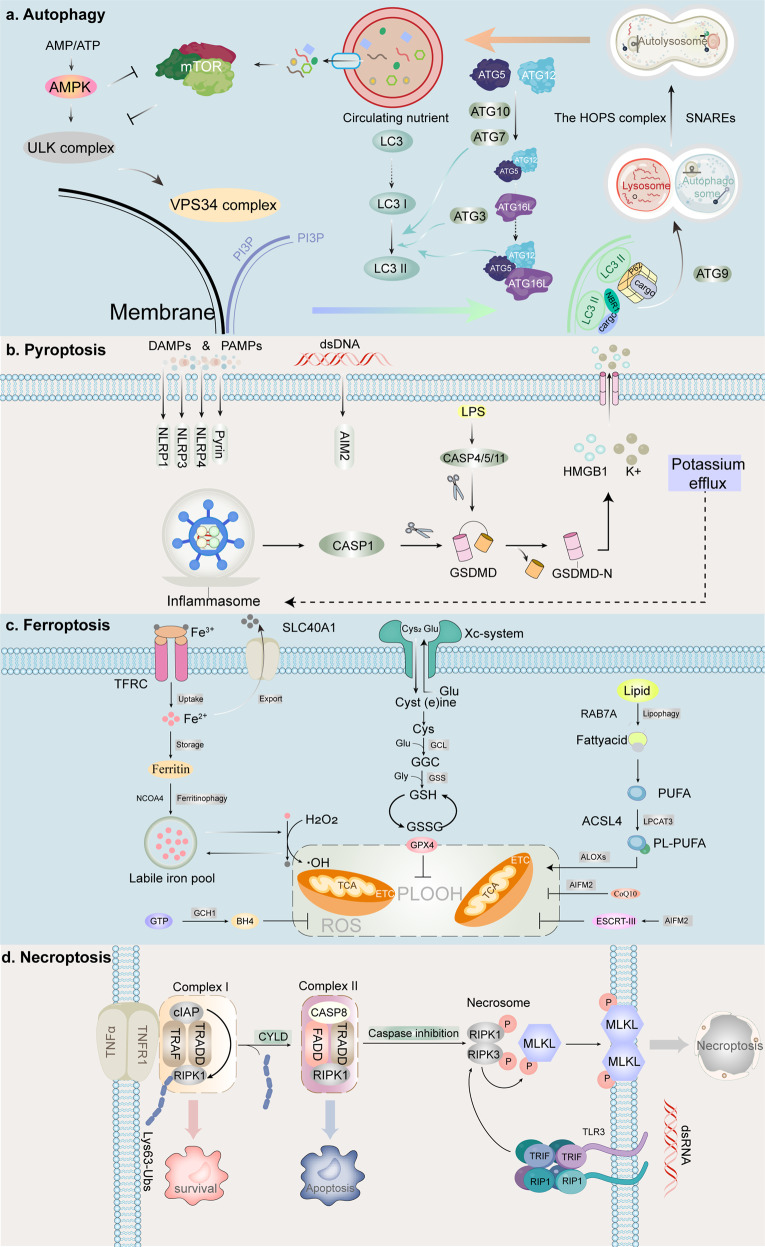
Core molecular mechanisms of autophagy, pyroptosis, ferroptosis, and necroptosis. **a** The ULK complex initiates autophagy by responding to nutrient stress signals from mTOR and energy stress signals from AMPK, which eventually activate VPS34. VPS34 complex generates PI3P at membrane to recruit and assemble ubiquitin-like coupling systems. In LC3 lipidation, ATG7, ATG3, and ATG5-ATG12-ATG16L complexes are ubiquitin enzymes that recruit loads to cargo receptors such as SQSTM1/P62 and NBR. In the presence of ATG9, the phagosome expands and eventually closes to form autophagosomes, which are subsequently fused with lysosomes to form autolysosomes mediated by SNAREs and the HOPS complex. Finally, cargoes are degraded by lysosomal hydrolases and nutrients are recycled. **b** Cytoplasmic sensor proteins such as NOD-like receptor family members (NLRP1, NLRP3, and NLRP4), AIM2 and Pyrin proteins are stimulated by PAMPs or DAMPs, recruiting and activating CASP1 via ASC. CASP4/5/11 are activated in the cytoplasm binding to LPS. Finally, activated CASP1 and CASP4/5/11 cause the cleavage and production of GSDMD-N, which leads to pyroptosis by activating typical and atypical inflammasomes. Pyroptosis regulated by potassium efflux triggers the release of HMGB1 and K+. **c** During ferroptosis, two fundamental processes trigger oxidative membrane damage: iron accumulation and lipid peroxidation. The transferrin–transferrin receptor (TF–TFRC) complex, iron export transporter, and ferritinophagy contribute to ferroptosis by increasing iron accumulation through increased iron uptake, restricted iron efflux, and decreased iron storage, respectively. The ACSL4–LPCAT3–ALOXs pathway plays a critical role in promoting ferroptosis by activating lipid peroxidation to produce PLOOH from PUFA with the involvement of RAB7A-dependent lipophagy. Several antioxidant systems such as Xc-system–GSH–GPX4, AIFM2-CoQ10, GTP-GCH1-BH4, or ESCRT-III membrane repair system inhibit lipid peroxidation. **d** After TNFα binds to the receptor, the intracellular tails of TNFR1 recruit multiple proteins to form Complex I. Lys63-linked polyubiquitination (Lys63-Ub) of RIP1 mediated by cIAP is essential for the survival pathway. Deubiquitination of RIP1 by CYLD promotes the conversion of Complex I to Complex II. When CASP8 is activated in complex II, apoptosis is initiated. When CASP8 is inhibited, MLKL, RIPK1, and RIPK3 are recruited to assemble the necrosome through phosphorylation. The phosphorylation-mediated activation of MLKL and subsequent MLKL-mediated membrane pore formation results in necroptosis

Cell death is classified into two categories based on the rate at which it occurs and whether drugs or genes may influence it: accidental cell death and regulated cell death (RCD).^1^ Accidental cell death results from the biological process, while RCD is mediated by signal transduction pathways and well-defined mechanisms of action.^1^ RCD plays a vital role in homeostasis maintenance and diseases development. Based on different morphological, biochemical, immunological, and genetic characteristics, RCD is subdivided into apoptotic and non-apoptotic categories.^2,3^ Non-apoptotic RCD can be subdivided into autophagy, ferroptosis, pyroptosis, and necroptosis (Table 1 and Fig. 1). Immunogenic cell deaths (ICD) mentioned in Table 1. will be described in detail below. Resistance to apoptosis is a general characteristic of cancer.^4^ Research on apoptosis has been conducted for more than 30 years. Nevertheless, therapeutic agents targeting apoptosis regulators such as apoptosis-related caspases or B-cell lymphoma-2 (BCL-2) family proteins have poor effects in antitumor therapy.^5^ On the contrary, non-apoptotic RCD affects the development of cancer and its response to therapy.^1–3^ For example, in genetic engineering mice, enhanced sensitivity of tumors to ferroptosis significantly inhibited the formation and progression of pancreatic cancer.^6^ The KRAS mutation-driven lung cancer model, however, suggests that autophagy is necessary for maintaining mitochondrial function and providing energy for cells to survive and grow.^7^ Inflammasome, a key component of pyroptosis, plays a critical role in chemoresistance in oral squamous cell carcinoma and insensitivity to radiotherapy in glioblastoma.^8,9^ Key mediators in the process of necroptosis are thought to promote head and neck squamous cell carcinoma metastasis and progression as well as negatively affect the prognosis in glioblastoma; however, necroptosis has also been reported to act as a defense mechanism, playing a tumor-suppressive role when tumor cell apoptosis is impaired in leukemia and colorectal cancer.^10–13^ Therefore, targeting non-apoptotic RCD has attracted much attention in the field of antitumor therapy.

**Table 1:** Morphological, biochemical, immune features and major regulators of autophagy, pyroptosis, ferroptosis, and necroptosis

Type	Morphological features	Biochemical features	Immune features	Major regulators
Autophagy	Autophagic vacuolization	Caspase-independent, LC3 lipidation, formation of autophagosome, elevated autophagic flux, and lysosomal activity	ICD	Positive: AMPK, ULK, VPS34 Negative: mTOR
Ferroptosis	Cells swelling, pore formation on cells membranes, smaller mitochondria, decreased mitochondria crista, and elevated mitochondrial membrane densities	Caspase-independent, iron accumulation, lipid peroxidation, Xc-system/GSH/GPX4 pathway inhibition	ICD	Positive: TFRC, ALOX, ACSL4, LPCAT3 Negative: GPX4, AIFM2, ESCRT-III
Pyroptosis	Cells swelling, pore formation on cells membranes, rupture, and bubbling of plasma membranes, moderate chromatin condensation	Caspase-dependent, gasdermin cleavage, formation of inflammasome, IL-18 and IL-1β release	ICD	Positive: CASP1, CASP4, CASP5, CASP11, Gasdermin Negative: ESCRT-III and GPX4
Necroptosis	Cells swelling, pore formation on cells membranes, plasma membranes rupture, and moderate chromatin condensation	Caspase-independent, RIPK1/RIPK3-mediated phosphorylation of MLKL, and the assembly of necrosome	ICD	Positive: RIPK1, RIPK3 and MLKL Negative: AURKA and ESCRT-III

The immune system contributes to preventing the occurrence, progression, and metastasis of tumor and regulating tumor response to therapy. Immune surveillance provides a way to identify, control and kill tumor cells.^14–16^ However, tumor cells evade immune surveillance by reducing immunogenicity and forming an immunosuppressive network.^14–16^ Immunotherapy harnesses the immune system against tumors by stimulating antitumor immune responses, including immune checkpoint inhibitors (ICIs),^17–19^ chimeric antigen receptor T cells (CAR-T cells),^20^ dendritic cell vaccines,^21^ and cytokine therapies.^22^ For the past few years, ICIs have made significant breakthroughs in the field of antitumor therapy.^23–25^ Mechanically, ICIs inhibit cancer development by restoring the function of effector T cells.^17,19^ Traditionally, it was believed that immunotherapy-activated CD8+ T cells induce tumor cell death mainly through the perforin-granzyme pathway and the Fas-Fas ligand (FASL) pathway.^26,27^ However, many studies have surprisingly revealed that CD8+ T cells can suppress tumors by inducing ferroptosis and pyroptosis.^28–31^ Similarly, recent studies have shown that non-apoptotic RCD participates in the survival, differentiation, activation, and translocation of immune cells and its function performance (both antitumor and tumor-promoting effect cells).^32,33^ Meanwhile, tumor-autonomous non-apoptotic RCD can affect tumor growth by modulating immune responses.^34,35^ It is worth stating that the process of tumor cell deaths that stimulates an adaptive immune response is called ICD.^36^ During ICD, damage-associated molecular patterns (DAMPs) including a variety of biomolecules including high mobility group box 1 (HMGB1), mitochondrial DNA, and ATP, and pathogen-associated molecular patterns (PAMPs) including various microbial pathogen components such as lipopolysaccharide (LPS) can be identifiable by pattern recognition receptors (PRRs) and play a dual role in tumor immunity.^37^ Besides the antitumor immune responses, the release of cytokines and chemokines facilitates the inflammatory responses that promote tumor growth.^38,39^ It is possible for ICD to occur in the context of autophagy, ferroptosis, pyroptosis, and necroptosis.^40–43^ We, therefore, hypothesize that these non-apoptotic RCD processes may be a double-edged sword for tumor immune responses.

In this context, we review autophagy, ferroptosis, pyroptosis, and necroptosis on tumor development, the multilevel relationships with tumor immune responses, and the critical roles in immunotherapy. In addition, we discuss the potential application of targeting non-apoptotic RCD to enhance the efficacy of immunotherapy in malignancy.

## Overview of autophagy

Eukaryotic cells utilize autophagy for maintaining homeostasis and managing lipid metabolism.^44,45^ Activated by various stress states, autophagic membrane structures are formed to engulf and degrade intracellular structures, including damaged organelles, unfolded proteins, and pathogens.^42,46^ Autophagy was initially thought to be a “bulk degradation” process. Still, new findings suggest that specific cargoes such as organelles and proteins can be recognized by selective autophagic receptors (SARs) and be degraded.^47,48^

The autophagy initiation is mediated by the unc-51-like kinase (ULK) complex,^49^ which shifts to an active state when mTOR complex 1 (mTORC1) is inhibited, or 5′-AMP-activated protein kinase (AMPK) is activated stimulated by stress signals then activating vacuolar protein sorting 34 (VPS34).^50,51^ The VPS34 complex acts as a phosphatidylinositol 3-phosphate kinase (PI3K) to generate phosphatidylinositol 3-phosphate (PI3P) which acts as a scaffold to recruit PI3P-binding molecules, forming an isolated pre-autophagosomal structure called phagosome.^52,53^ Specifically, PI3P recruits and assembles two ubiquitin-like coupling systems that are involved in LC3 lipidation and autophagosome formation.^53–55^ During LC3 lipidation, LC3 is sheared to the soluble form LC3I as the precursor of LC3II which is a docking site covalently attaching to the membrane of phagosomes for cargo receptors.^48,56,57^ The receptor binds to specific cargoes through ubiquitin labeling, which is central to the selective recruitment of loads during autophagy.^48,57^ Subsequently, the phagosome extends and eventually closes to form a separate compartment called autophagosome.^58,59^ Autophagosomes are transported to the perinuclear region, where they fuse with proximal lysosomes to form autolysosomes.^60,61^ In the presence of lysosomal hydrolases, cargoes are degraded, and nutrients are recycled.^62^

The dysregulation of autophagy contributes to tumor growth and progression. Studies in 1999 showed that a single allele of Beclin1 was absent in 40–75% of disseminated human breast and ovarian cancers, which was the first time that autophagy was reported to be associated with human cancer.^63,64^ Similarly, a heterozygous deletion of ATG5 at chromosome 6q21 is a prominent feature of advanced melanoma in humans and affects KRAS-driven pancreatic tumor development and metastasis.^65,66^ In addition to effects on tumor cells, autophagy defects can indirectly promote tumorigenesis through inflammation.^67^ The Thr300Ala mutation in ATG16L1 may lead to chronic inflammatory Crohn’s disease, thereby predisposing patients to colorectal cancer.^68^ Interestingly, a KRAS mutation-driven pancreatic cancer model revealed that tumor growth is facilitated by autophagy in the cancer mesenchymal region. Pancreatic stellate cells secrete alanine through autophagy by pancreatic tumor cells to promote growth-friendly mitochondrial metabolism.^69^

## Overview of ferroptosis

The term ferroptosis introduced in 2012 refers to iron-dependent RCD caused by the excessive amount of lipid peroxidation, resulting in the ruptured plasma membrane.^70^ When ferroptosis occurs, iron accumulation and lipid peroxidation both contribute to oxidative membrane damage.^71,72^ Increased iron accumulation is a key trigger of ferroptosis in animal models.^73^ Specifically, transferrin promotes ferroptosis by mediating iron uptake through the transferrin receptor (TFRC).^74,75^ Degrading intracellular iron storage proteins or iron export transporter solute carrier family 40 member 1 (SLC40A1) by the autophagy increases iron accumulation, thereby initiating or enhancing ferroptosis.^76–78^

Excess intracellular iron can contribute to subsequent lipid peroxidation through the production of reactive oxygen species (ROS) and the activation of iron-containing enzymes such as arachidonic acid lipoxygenases (ALOXs).^79–81^ In the presence of long-chain fatty acid–CoA ligase 4 (ACSL4) and lysophospholipid acyltransferase 5 (LPCAT3), polyunsaturated fatty acid (PUFA) is catalyzed to develop phospholipids-polyunsaturated fatty acid (PL-PUFA).^82,83^ Finally, PL-PUFA is mediated by ALOXs to produce phospholipid hydroperoxides (PL-PUFA-OOH), which can promote ferroptosis.^84^ Ferroptosis is primarily a process of balancing oxidative and antioxidant damage.^85^ Glutathione (GSH)-glutathione peroxidase 4 (GPX4) antioxidant system plays an essential role in protecting cells from ferroptosis. Xc-system is responsible for the import of cyst(e)ine as a rate-limiting substrate for GSH synthesis in exchange for intracellular glutamate (Glu).^71^ GPX4 uses GSH as a reducing cofactor that reduces PLOOH to fatty alcohol, thereby inhibiting ferroptosis in tumor cells.^86–88^ Other antioxidant systems, such as the coenzyme apoptosis-inducing factor mitochondrial 2-coenzyme Q10 (AIFM2-Q10),^89^ tetrahydrobiopterin (BH),^90^ as well as sorting complexes in the endosomes as a requirement for transport III (ESCRT-III) membrane repair system,^91^ all contribute to antagonize ferroptosis in solid tumors.^92^ Ferroptosis was initially regarded as the cell death process that did not depend on autophagy.^70^ However, recent studies have revealed that iron accumulation and lipid peroxidation are promoted by excessive activation of selective autophagy, resulting in ferroptosis.^93,94^ Selective autophagy mainly includes nuclear receptor coactivator 4 (NCOA4)-induced ferritinophagy,^95,96^ heat shock protein 90 (HSP90)-regulated chaperone protein-dependent autophagy,^97^ RAS oncogene family member RAB7A-mediated lipophagy,^98^ and clockophagy associated with SQSTM1,^99^ respectively, to selectively degrade ferritin, GPX4, lipid droplets, thereby increasing intracellular iron and free fatty acid levels and accelerating the peroxidation of lipids to promote ferroptosis.

It has been progressively recognized that several oncogenic pathways are closely associated with ferroptosis.^100^ For example, most KRAS mutation-driven pancreatic cancers are sensitive to ferroptosis activators, Erastin.^101,102^ Furthermore, new evidence suggests that as cancer suppressor gene, p53 inhibits cyst(e)ine uptake and sensitizes cells to ferroptosis by suppressing the expression of Xc-system.^81,103,104^ However, it has also been shown that p53 can limit Erastin-induced ferroptosis in a transcription-independent manner by blocking dipeptidyl peptidase-4 (DPP-4) activity.^105^

## Overview of pyroptosis

Pyroptosis is an ICD caused by caspases found in immune cells during microbial infections.^106^ Inflammasome-associated caspases, such as CASP1, CASP4, CASP5, and CASP11 (mouse), are mainly responsible for regulating pyroptosis,^107,108^ whereas some caspases associated with apoptosis such as CASP3^109^ and CASP8^110^ also play a role in pyroptosis. And the cleavage of gasdermin (GSDM) family members such as GSDMD^110^ and GSDME^109^ mediated by caspase is crucial to trigger pyroptosis. In typical and atypical pyroptosis pathways, CASP1/4/5/11 has been reported for GSDMD cleavage.^107,108^ Under particular circumstances, apoptosis-dependent CASP8 can directly cleave GSDMD, which triggers pyroptosis.^110^ CASP8-dependent cleavage of GSDMD promotes host defense against infection while also enhances tumor necrosis factor (TNF) lethality.^111^ In addition, GSDME can be cleaved by CASP3/8, thereby converting non-inflammatory apoptosis to pyroptosis. Granzyme B (GZMB) acts at the same site to cleave GSDME, activating caspase-independent pyroptosis in target cells.^31,112^ Similarly, GSDMB is cleaved by CASP1^113^ or granzyme A (GZMA).^114^ Here is not detailed explanation of GSDMA/C-mediated pyroptosis.^115^

In the typical inflammasome activation pathway, PAMPs or DAMPs are detected by cytoplasmic sensor proteins such as NOD-like receptor family members (NLRP), absent in melanoma 2 (AIM2), and Pyrin proteins.^116–119^ For example, AIM2 are activated by detecting and then binding precisely to cytoplasmic double-stranded DNA.^116^ NLRP3 responds to components such as ATP, crystals, and viruses, causing potassium efflux to trigger NLRP3 activity.^120,121^ Activated sensor proteins recruit and activate CASP1 via apoptosis-associated speck-like protein containing a CARD (ASC) which together constitute the inflammasome.^117^ In the atypical inflammasome activation pathway, CASP11 in mice or CASP4/5 in humans is activated in cytoplasm binding directly to LPS.^108^ Finally, CASP1 or CASP4/5/11 causes the release of active GSDMD N-terminal fragment (GSDMD-N) which binds to acidic phospholipids on the plasma membrane and forms oligomeric death-inducing pores, increasing intracellular osmolality thus leading to cytolysis to mediate pyroptosis.^122–124^

Pyroptosis appears to play a dual role in tumor development, either promoting tumor or causing tumor regression which depends on the context in which tumor cells are located. For example, in pancreatic cancer cells, macrophage-stimulating factor 1 (MST1) promotes CASP1-dependent pyroptosis by inducing the production of ROS.^125^ Gao et al. have shown that the levels of GSDMD protein were extremely increased in NSCLC. High GSDMD expression was associated with aggressiveness of NSCLC, including larger tumor volume and higher TNM stage.^126^ Nevertheless, activation of pyroptosis can also induce potent antitumor activity.^127^ For example, in hepatocellular carcinoma (HCC) cells, pyroptosis induced by NLRP3 inflammasomes significantly impedes tumor growth characteristics and metastatic potential.^128^ Aside from the digestive system,^129,130^ pyroptosis acts an equally important part in the development of cancers in respiratory,^131^ reproductive,^132^ and hematopoietic systems.^133^

## Overview of necroptosis

Necroptosis, introduced in 2005 by Degterev et al. is another form of ICD in which specific death receptors (DRs) including FAS and tumor necrosis factor receptor 1 (TNFR1), etc. or PRRs such as toll-like receptor3 (TLR3) recognize unfavorable signals from the intra- and extra-cellular microenvironment to initiate necroptosis.^134–136^ Necroptosis, triggered by the same stimuli as apoptosis, is similar to necrosis in its morphology (e.g., organelle swelling and ruptured plasma membrane).^137,138^ Necroptosis appears to be a backup mechanism of apoptosis, in which a key component of necroptosis, necrosome, assembles in TNFR1 stimulation in response to viral infection when CASP8 involved in apoptosis is inhibited.^139,140^ Moreover, the reduction of intracellular ATP occurs during the transition from apoptosis to necroptosis or necrosis.^141^

In response to TNFα, the intracellular tails of TNFR1 recruit a variety of proteins that together form a signaling complex called “Complex I” in which the ubiquitination of RIPK1 is regulated by cellular inhibitor of apoptosis protein (cIAP), which is indispensable for nuclear factor kappa-B (NF-κB) and MAPK activation involved in the survival pathway.^142–145^ The conversion of Complex I into Complex II is facilitated by deubiquitination of RIPK1 by cylindromatosis (CYLD).^142^ When CASP8 is activated in complex II, apoptosis is initiated.^143,146^ However, in RIPK3-rich cells, when CASP8 is inhibited, intracellular junctional molecules sequentially recruit RIPK1, RIPK3, and mixed lineage kinase domain-like (MLKL) to complete necrosome assembly after phosphorylation events.^147–149^ RIPK3 can also be activated when TLR3 is sensed by double-stranded RNA (dsRNA) in the endosome or ZBP1 is sensed by cytosolic DNA.^135,150^ When MLKL is activated by RIPK3, oligomerization and subsequent translocation occurs. As a result, plasma membrane permeability increases, causing membrane rupture and the release of DAMPs.^151^

There has been evidence to suggest that necroptosis acts as a tumor suppressor in most cases.^152,153^ Two-thirds of samples in a study of more than 60 cancer cell lines showed decreased levels of RIPK3, which indicates that the cancer cells prefer to escape necroptosis and survive. Furthermore, necroptosis is strongly associated with cancer prognosis. The Cox proportional risk model showed that the expression of RIPK3 is an independent prognostic factor in colorectal cancer patients with regards to overall survival and disease-free survival.^154^ Recently, a study has shown that the expression of RIPK1, RIPK3, and MLKL was linked to better overall survival in HCC.^155^ Furthermore, methylation near the transcription start site silences RIPK3 expression in cancer cells. Therefore, hypomethylation drug treatment can improve prognosis by restoring RIPK3 expression and increasing sensitivity to chemotherapeutic agents.^156^

## Autophagy, ferroptosis, pyroptosis, and necroptosis synergize antitumor immune response

**Fig. 2:**
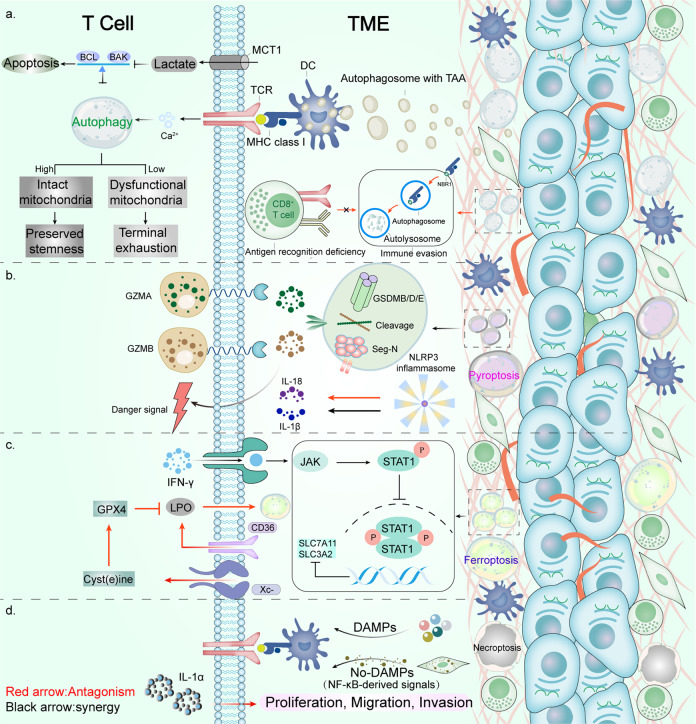
Crosstalk between T cells and dying cancer cells in the tumor microenvironment. **a** In dead cancer cells, autophagy increases the production of autophagosomes with TAA, which promotes DC-mediated cross-presentation. When TCR is stimulated, activated T cells have enhanced levels of autophagy, which is linked to rapidly increased calcium levels. By reprogramming metabolic pathways, autophagy is vital for mitochondrial integrity, which maintains T cells’ homeostasis. High levels of lactate in tumors inhibit autophagy and induce apoptosis in naive T cells. Furthermore, NBR1-mediated MHCI degradation through autophagy reduces MHCI expression on the surface of cancer cells and impairs CD8+ T cells recognition of antigens. **b** On the one hand, tumor cells via pyroptosis pathway facilitate the recruitment of CD8+ T cells by releasing danger signals. On the other hand, CD8+ T cells induce cancer cell pyroptosis by secreting GZMA and GZMB, which can cleave GSDMB/D/E. NLRP3 inflammasomes promote IL-18 and IL-1β secretion, which have tumor-promoting or antitumor effect dependent on the context of TME. **c** Significant lipid peroxidation activity can occur in CD36-positive CD8+ T cells, which results in ferroptosis induced by GPX4 inhibitors, leading to reduced release of IFN-γ. IFN-γ released by CD8+ T cells induces tumor cells ferroptosis through the activation of JAK1-STAT1 signaling, which transcriptionally regulates the expression of Xc- component, SLC7A11and SLC3A2. **d** Two strategies have been reported to trigger antitumor immunity through necroptosis. (1) DAMPs released from tumor cells through necroptosis promote cross-priming of DCs, and subsequent cytotoxic effects of CD8+ T cells. (2) Fibroblasts in the TME through necroptosis induce the robust immune response via NF-κB signaling. Besides, the necroptosis-induced release of regulatory cytokines such as IL-1α by CD8+ T cells triggers inflammation and promotes tumor growth by facilitating proliferation and migration of cancer cells

**Fig. 3:**
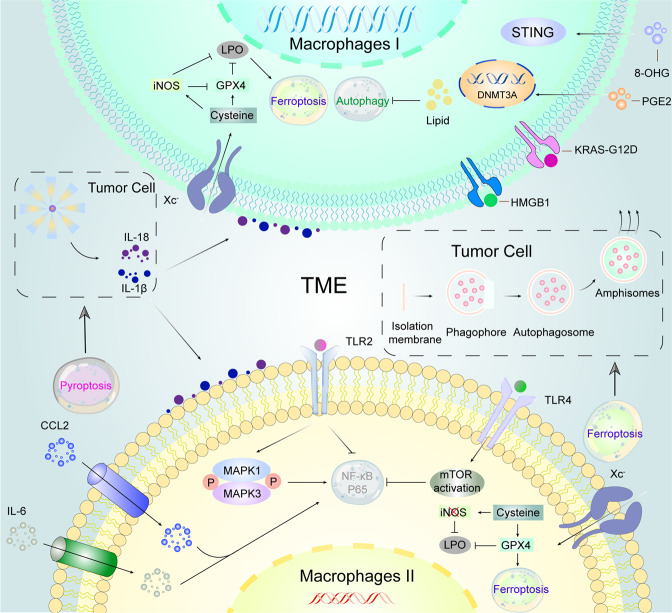
Crosstalk between Macrophages I/II cells and dying cancer cells in the tumor microenvironment. Tumor cells affect the function of macrophages by releasing DAMPs such as KRAS-G12D, HMGB1, 8-OHG, and PGE2 through ferroptosis. Pancreatic cancer cells can release KRAS-G12D during ferroptosis, whose exocytosis is largely dependent on autophagosome-derived amphisomes. KRAS-G12D triggers M2 cells polarization by binding to AGER which might induce adaptive immunosuppression. In addition, iron-addicted cancer cells activate STING-dependent DNA sensor pathways in M1 cells through the release of 8-OHG to create an inflammatory microenvironment for tumor growth. Similarly, PGE2, induced by ferroptotic cancer cells can act on DNMT3A, causing DNA methylation thus suppressing immunogenic genes. M1 cells are more resistant to ferroptosis than M2 cells, even in the absence of GPX4. Mechanically, iNOS which is highly expressed in M1 cells but inhibited in M2 cells produces more NO•, replacing GPX4 as a negative regulator of ferroptosis. Furthermore, excessive lipid accumulation in macrophages can prevent autophagy in obese mice, thus promoting the conversion of macrophages into pro-inflammatory M1 cells. In the TME, IL-6, and CCL2 trigger autophagy by binding to IL-6R and CCR2, respectively, which is essential for macrophage polarization to M2 phenotype. Furthermore, in M2 cells, TLR2 signaling inhibits the NF-κB signaling pathway through selective autophagy. TLR2 signaling also promotes sustained phosphorylation of MAPK1 and MAPK3, which stimulates autophagy-dependent NF-κB regulation. Autophagy can be inhibited in M2 cells regulated by TLR4-mTOR pathway. In addition, triggered by NLRP3 inflammasome, tumor cell-derived IL-1β and IL-18 recruit M1/2 cells to inhibit or promote tumor progression

**Fig. 4:**
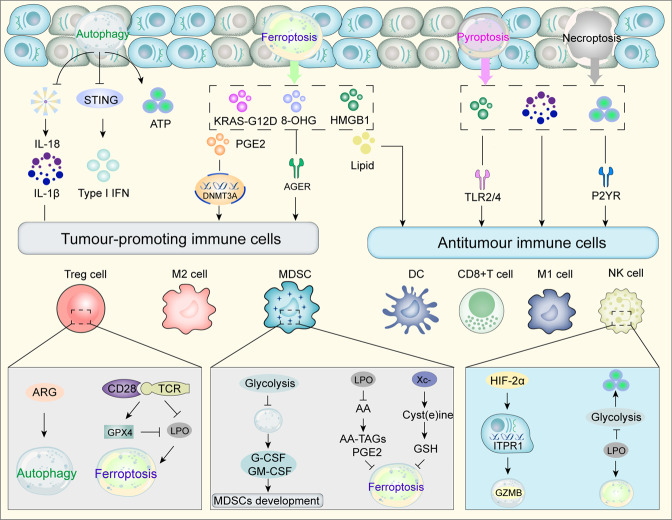
The role of autophagy, ferroptosis, pyroptosis, and necroptosis in immunogenic cell death. During ICD, cancer cells can release specific DAMPs such as HMGB1, ATP, lipid mediator, etc. and cytokines such as IL-18, IL-1β, etc. through specific cell death such as autophagy, pyroptosis, ferroptosis, and necroptosis to act on tumor-promoting immune cells including M2, MDSCs and Treg cells or antitumor immune cells including M1, NK, and CD8+ T cells by binding to receptor specifically. It’s worth noting that mitophagy, a selective form of autophagy removes damaged mitochondria, suppresses type I IFN production and inhibits inflammasome activation thus reducing IL­1β and IL­18 production. The occurrence of glycolysis in MDSCs reduces autophagy, which reduces the expression of G-CSF and GM-CSF and prevents MDSCs proliferation. And MDSCs selectively accumulate AA-tags and PGE2 but not PL-PUFA and LPOs, thus forming ferroptosis resistance. Moreover, MDSCs with high Xc-system expression consume cyst(e)ine which is not transported to the microenvironment due to the absence of ASC transporter proteins, thereby depriving of the cyst(e)ine required for T cells activation. Tregs express high levels of ARG2, leading to activation of autophagy. When TCR/CD28 is co-stimulated, ferroptosis is reduced by the expression of GPX4 in Tregs. In addition, hypoxia induces HIF-2 to transport to the nucleus and activates the autophagy sensor ITPR1 to degrade NK cell-derived GZMB. Furthermore, lipid peroxidation in ferroptosis inhibits glucose metabolism in NK cells leading to NK cells dysfunction

The organism can initiate autophagy, ferroptosis, pyroptosis, and necroptosis as defense in the face of various intra- and extra-cellular stress stimuli, acting to inhibit the proliferation of cancer cells. This defense is achieved in large part through a synergistic antitumor immune response. Specifically, non-apoptotic RCD is involved in the survival, differentiation, activation, transport, and functional performance of immune cells (both antitumor and tumor-promoting effect cells). Meanwhile, tumor-autonomous non-apoptotic RCD can alter tumor growth by modulating immune responses (Figs. 2, 3, and 4).

## Autophagy synergizes antitumor immune response

Recent research has shown that autophagy contributes to antitumor immunity such as innate immunity, antigen presentation, immune cell development, and inhibition of immune evasion.^157^ Autophagy substrates, DAMPs and PAMPs trigger innate immunity whose elimination through autophagy is necessary for immune homeostasis to protect the cells from exposed membranes and other organelles.^158^ For example, mitophagy is a selective form of autophagy in response to diverse stimuli that removes damaged mitochondria, suppresses type I IFN production, and inhibits inflammasome activation thus reducing the production of IL­1β and IL­18 as a result of preventing the accumulation of mitochondrial-derived DAMPs, such as ROS and mitochondrial DNA (mtDNA).^159^ Autophagy inactivation increases the production and secretion of inflammatory cytokines such as type I, II IFNs and TNFα. As well, mice with essential autophagy genes produce fewer type I and II IFNs, TNFα, and C-C Motif Chemokine Ligand 2 (CCL2).^160,161^ Therefore, we may conclude that even though autophagy-deficient primary tumors infiltrated with the pro-inflammatory TME may be suppressed, induction of inflammation-related cancer probably results from autophagy deficiency and inability to remove bacteria, organelles, and damaged proteins.^162^ In autophagy-deficient mice, unchecked innate immune activation and damage in normal tissues leads to human diseases related to defective autophagy genes, including Crohn’s disease, the risk factor for colon cancer.^159^

Autophagy serves as an ICD that promotes ATP secretion by facilitating the migration of ATP-containing lysosomes toward the plasma membrane.^163^ Due to ATP’s crucial role as a chemotactic signal, chemotherapeutic agents are less likely to trigger a robust antitumor immune response when autophagy is lost in tumor cells.^164^ Besides ATP, other signals from the intra- and extra-cellular can be presented to antigen-presenting cells (APCs) through autophagy. Pathogens are engulfed by autophagosomes and degradation products are delivered to the major histocompatibility complex II (MHCII) of APCs, thereby activating CD4+ T cells.^165^ A study has concluded that when ATG5 is defective, the fusion of lysosomes with phagosomes is delayed, thereby inhibiting antigen presentation by dendritic cells (DCs) via the MHCII and activation of CD4+ T cells.^166,167^ Autophagy may also promote the presentation of extracellular antigens to MHCII through ATG8/ LC3-related phagocytosis (LAP), one of the atypical autophagy pathways. LAP participates in the uptake and degradation of dying cells by macrophages, which subsequently present antigens to immune effector cells. In the absence of LAP, inflammation is caused by an imbalance of pro-inflammatory and anti-inflammatory cytokines.^168–170^ Furthermore, autophagy contributes to MHCI-mediated cross-antigen presentation. α-Tocopherylacetic acid (α-TEA) induces autophagy and produces autophagosome-rich supernatant fraction, α-TAGS, which acts as a carrier of antigen for cross-presentation to specific CD8+ T cells via MHCI.^171,172^

Studies have demonstrated that autophagy is closely related to T cells survival, activation, proliferation, differentiation, and functional performance.^173^ The survival of peripheral naive T cells is dependent on T cell receptor (TCR) interaction with stromal cells and the process of IL-7 signaling, which is involved with ATG3-dependent autophagy proteins.^174^ On the contrary, in tumor-bearing mice, tumor-infiltrating T cells (especially naive T cells) often exhibit impaired autophagy and undergo apoptosis, thus supporting tumor immune escape which is caused by tumor-derived lactate suppressing FIP200 expression in T cells by disrupting the balance between pro-and anti-apoptotic BCL2 family members.^175^ Once TCR is stimulated, activated T cells have enhanced levels of autophagy, which is associated with rapid elevation of calcium levels that activate ULK1 complex phosphorylated by AMPK to promote autophagy.^176,177^ When ATG3, 5, and 7 genes are defective in activated T cells, cyclin-dependent kinase inhibitor1B (CDKN1B) cannot be degraded, resulting in the inability of T cells to proliferate efficiently.^178^ Autophagy regulates T cells differentiation by affecting different metabolic programs.^179,180^ When activated T cells are more likely to induce mTOR, T cells differentiate into T helper cells (Th cells) due to enhanced glycolysis. When the activated level of AMPK is higher, primitive T cells undergo lipid peroxidation and preferentially differentiate into T regulatory cells (Treg cells).^181^ In addition, autophagy maintains T cells homeostasis by regulating the mitochondria content during T cells development. Defective autophagy leads to inadequate degradation of mitochondria components and increases ROS production thereby disrupting T cells development and function.^182^ Interestingly, lipophagy may be involved in the fatty acid β-oxidation process and promote the formation of memory T cells.^183^ The effector CD8+ T cell that lacks autophagy is incapable of establishing a lasting memory for providing antitumor immunity.^179,184^

In addition to contributing to the antitumor immune effect of T cells, autophagy is involved in B cells development, differentiation, and antibody production. It was demonstrated that ATG5 was required for effector B cells development and maintenance of B1 CD5+ (B1a) cell numbers. Knockdown of ATG5 resulted in impaired development of B cells in the bone marrow and reduced numbers of B1a cells in the peripheral blood.^185^ On the contrary, another study showed that autophagy was not necessary for the transition of progenitor B cells to pre-B cells and B cells activation, but was required for plasmocyte differentiation and specific production of IgM and IgG in response to LPS stimulation.^186^ The tumor-derived autophagosomes (DRibbles) stimulate the activation of B cells, which secrete antibodies and cytokines.^187^ Mitochondrial autophagy is required to maintain the survival and function of reactive B memory cells. Mice lacking mitochondrial autophagy genes accumulate mitochondria and experience oxidative phosphorylation and fatty acid synthesis, leading to the loss of B memory cells.^188^ Similarly, autophagy facilitates the differentiation of monocytes to macrophages stimulated by colony-stimulating factor 1 (CSF1) and CSF2. Mechanically, CSF1 promotes autophagy by increasing the expression and phosphorylation of ULK1.^189^ CSF2 helps Beclin1 release from Bcl-2 protein and thus stimulates autophagy by activating c-Jun N-terminal kinase (JNK) and blocking ATG5 cleavage.^190^ Furthermore, defective autophagy promotes inflammation by promoting M1 polarization. Recent evidence suggests that in obese mice, excessive lipid accumulation in macrophages can promote the conversion of macrophages to pro-inflammatory M1 cells via inhibited autophagy pathway, leading to the progression of liver inflammation and liver injury.^191^

As we know, cancer immunotherapy can be improved by blocking PD-1/PD-L1 immune checkpoints by binding PD-L1 on cancer cells to PD-1 on T cells resulting in T cells inactivation, and consequently cancer immune invasion.^192,193^ A growing body of evidence suggests autophagy may affect cancer cells’ immune escape through the degradation of immune checkpoint protein. A recent study has shown that as autophagy receptor for PD-L1 binding, Huntingtin-interacting protein 1-related (HIP1R) induces PD-L1 degradation in lysosomes, subsequently suppressing the tumor growth via activation of T cells.^194^ However, cancer cells inhibit the degradation of PD-L1 by autophagy via transcriptional modification. For example, in a breast tumor model, epidermal growth factor receptor (EGFR)/β1,3-N-acetylglucosaminyltransferase-3 (B3GNT3) pathway-mediated PD-L1 glycosylation inhibits autophagic degradation of PD-L1, leading to tumor immune escape.^195^ Likewise, a colon tumor model shows that palmitoylation of acyl transferase DHHC3-induced PD-L1 decreases its autophagic degradation, causing the immune suppression and tumor growth.^196^ In addition to the modification of PD-L1, the cell membrane chemokine-like factor super family 6 (CMTM6) binds to PD-L1, inhibits endocytosed degradation of PD-L1, leading to tumor immunity evasion.^197^ Nevertheless, another study demonstrates that the activation of autophagy increases the expression of PD-L1 by 5-hydroxytryptamine receptor 1A (5-HT1AR)/autophagy/STAT3 phosphorylation pathway in lung cancer patients suffering from depression that results in immune escape which remains to be determined.^198^ As another immune tolerance checkpoint, cytolytic T lymphocyte-associated antigen-4 (CTLA-4) is an effectively therapeutic target for cancer patients. In the presence of CTLA-4, PI3K/AKT/mTOR pathways are activated significantly and translocation of forkhead box protein O1 (FOXO1) to the nucleus is induced, which constrains LC3β transcription and autophagosomes formation, consequently inducing autophagy deficiency.^199^ Nevertheless, the activation of autophagy can increase CTLA-4 expression, restore CTLA-4 suppressor activity and expand Tregs which can inhibit inflammation and suppress inflammatory cancer.^200^

As another immunologic tolerance molecule, indoleamine 2,3 dioxygenase (IDO) induced by tumor cells, tumor-associated myeloid-derived suppressor cells (MDSCs), and tumor-associated macrophages (TAMs) have been shown to suppress the CTLs responses and inflammatory DCs maturation, augment tolerogenic APCs, and stimulate Tregs differentiation, thereby alleviating effective antitumor immunity, facilitating immunological tolerance, and promoting the tumor growth.^174^ Inflammation-mediated IDO production can be inhibited by suppressing inflammation via autophagy.^201^ In turn, IDO can inhibit expression of mTOR, leading to autophagy via LC3 production. General control nonderepressible 2 (GCN2), recognized as a key effector of the IDO pathway inhibits the translation of initiation factor 2α (eIF2α), reduces protein synthesis, and blocks cell growth which are important in inflammatory carcinogenesis.^202^ There is a possibility that the IDO1/GCN2 autophagy pathway may play a significant role in human inflammatory conditions, since autophagy induced by IDO or GCN2 can protect organisms from death-causing inflammatory disorders.^203^

As well as PD-1/PD-L1, CTLA4, and IDO, SIRPα/CD47 immune checkpoints act as “don’t eat me” signals to prevent macrophage phagocytosis of cancer cells.^204,205^ CD47, which is highly expressed on cancer cells, binds to SIRPα on macrophages, inhibiting phagocytosis.^204^ Exosomal CD47 can inhibit pancreatic cancer cells from being cleared by phagocytes.^206^ It’s worth noting that PD-L1 or CD47 can be released by exosomes, cellular secreted vesicles (30–150 nm) with double-layer membrane not degraded by lysosomes, which is key to regulate crosstalk between cells.^207^ However, the relationship between exosomes and autophagy is still unclear, which requires further investigation.

## Pyroptosis synergizes antitumor immune response

Two concurrently published studies have found that tumor cells released danger signals that recruited antitumor immune cells through pyroptosis while immune cells induced pyroptosis in tumor cells, thereby establishing a positive feedback loop.^29,31^ A bioorthogonal system was built to release GSDMA3 into tumor cells and Wang et al. found that only 15% of tumor cells required pyroptosis to eliminate the whole tumor. Further studies concluded that the number of CD4+ T, CD8+ T, natural killer cells (NK cells), and M1 macrophages increased in tumors that underwent pyroptosis, while the number of monocytes, neutrophils, MDSCs, and M2 macrophages decreased.^29^ Along with increased levels of IL-1β, IL-18, and HMGB1, many effector genes for immunostimulatory and antitumor effects were upregulated, whereas various effector genes for immunosuppressive and tumor-promoting effects were downregulated.^29^

Zhang et al. reported that CD8+ T cells and NK cells induce pyroptosis of tumor cells independent of caspases through the GSDME-GZMB axis in their study.^31^ Recent studies have shown CD8+ T cells and NK cells can evoke tumor pyroptosis through the GSDMB-GZMA axis, which is induced by interferon-γ(IFNγ). GZMA may be delivered by immune cells to GSDMB-expressing cancer cells to promote antitumor immunity.^114^ In addition, a previous study showed that GSDMD plays a key role in antitumor function of CD8+ T cells.^131^ GSDMD and GZMB coexist near immune synapse and GSDMD deficiency has been shown to reduce the cell-killing capacity of CD8+ T cells. Considering that release of cytotoxic molecules into immune synapse is a key pathway for CTLs killing capacity, we hypothesize that GSDMD-GZMB axis may be a potential mechanism for CTLs to exert cytotoxicity. In the past, perforin has been thought to be the only protein responsible for pore formation on CD8+ T cells,^208^ but it was suggested by the authors that GSDMD could be a new pore-forming protein utilized by effector T cells to form pores in tumor cells.^40^ Nevertheless, the mechanism of GSDMD transportation from CD8+ T cells to tumor cells remains to be further explored.

Moreover, nuclear PD-L1 is able to modulate the non-canonical pyroptosis pathway mediated by GSDMC/CASP8 to induce tumor necrosis in cancer cells in hypoxic conditions. The nuclear PD-L1 family can switch TNFα-induced apoptosis into pyroptosis by upregulating GSDMC expression, leading to tumor necrosis and promoting tumor growth.^209^ Under hypoxic stress, the phosphorylated form of STAT3 interacts with PD-L1 to promote nuclear translocation of PD-L1, which in turn activates mRNA transcription of the GSDMC gene. In addition, TNFα treatment cleaves GSDMC via CASP8, releasing its N-terminal domain from the cell membrane and causing pyroptosis to occur.^209^

Together with the important role of GSDM family proteins in antitumor immune responses, inflammasomes are also key players. The antitumor role of inflammasomes in colitis-associated cancers has been extensively studied, and NLRP3 inflammasomes promote IL-18 secretion by bone marrow-derived cells and intestinal epithelial cells, thereby enhancing the activity of NK cells and CD4+ T cells to protect enterocytes from drug-induced damage in early colitis.^210,211^ Similarly, IL-18 induced through NLRP3 inflammasomes promote hepatic NK cell maturation, expression of the death ligand FasL, and lethality in tumors sensitive to FasL, thus inhibiting liver metastasis of colorectal cancer.^212^ Consistent with this, monocytes can be differentiated into DCs and maturation of DCs occurs through IL-1β induced by pyroptosis. What’s more, IL-1β can hyperactive DCs to facilitate tumor lysates as immunogens and bind to the surface of lymphocytes to drive antigen-specific cytotoxic CD8+ T cells responses.^213,214^ IL-1 has been shown to be effective in regressing different types of transplanted syngeneic tumors, as shown in the above research.^215–217^ In addition to its therapeutic effects, Allen and colleagues demonstrated that IL-1β protected against chemically induced colitis and colon cancer in animal models.^218^ Regardless, despite the fact that recombinant IL-1 has been shown to exert antitumor effects in various mouse studies, its systemic application has only produced limited benefits and significant toxicity on hematologic and solid tumors in a number of clinical trials.^219^ To prevent intense cytotoxicity, IL-1 is encapsulated into microspheres which is preferentially internalized by macrophages, thus promoting APCs activation.^220^ A system that delivers IL-1 intratumorally into fibrosarcoma-burdened mice can effectively cause tumor cell necrosis as well as strong leukocyte infiltration, which delays tumor growth.^220^ Furthermore, the release of IL-6 from pyroptotic cells contributes to the adaptive response by increasing cell trafficking, differentiation, and antibody production of CD8+ T cells, inhibiting Tregs differentiation and macrophages death.^221,222^

Interestingly, it is increasingly recognized that members of the intracellular sensor protein NLR family act independently of inflammasomes.^223,224^ Janowski et al. found that NLRC4 in TAMs inhibits melanoma progression by enhancing T cells function. When NLRC4 is defective in mice, macrophages are less able to produce cytokines and chemokines, and subsequently less able to recruit T cells near the tumor, which promotes tumor growth, independent of the inflammasome components ASC and CASP1.^225^

Along with inflammatory cytokines, the DNA binding protein, HMGB1, is also released during pyroptosis as well as necroptosis. Once released, HMGB1 binds to the RAGE receptors on tumor or immune cells, or TLR2/4 on the surface of immune cells.^226,227^ It’s worth noting that differential effects of these receptors on tumor growth are evident. HMGB1 through pyroptosis mediated by GSDME in epithelial cells, binds to RAGE and activates the extracellular regulated protein kinases (ERK1/2) signaling increasing cell migration by activating Rac1 and Cdc42.^228^ It has been reported that an elevated HMGB1 level is associated with invasion and metastasis in many cancer types. The inhibition of tumor growth by blocking HMGB1 and RAGE signaling was observed in a murine lung cancer model.^229^ In addition to RAGE receptors, HMGB1 signaling acts on neutrophils, monocytes, macrophages, etc. through TLR2 and TLR4 receptors activating transcription factors NF-κB and AP-1, triggering inflammation and cytokines’ production such as IL-6, TNFα, IL-8 needed for CD8+ T cells activation.^226^ Through HMGB1 signaling, chemotherapy and radiotherapy-induced cell death leads to an increase in antigen processing and cross-presentation on DCs.^230^ Thus, we may conclude HMGB1 can play a dual role by signaling through RAGE or TLRs in tumor growth.

As cells undergo pyroptosis and necroptosis, another DAMP, ATP released from cells, binds mainly to the purinergic P2X and P2Y receptors, exerting different antitumor effects depending upon each receptor.^231^ As a result of P2YR signaling, IL-8 is secreted,^232^ increasing neutrophil recruitment and phagocytosis.^233^ ATP binds to its purinergic receptor P2RX7, activates NLRP3 inflammasomes on myeloid APCs, and stimulates IL-1 signaling.^234^ A recent study found that mice lacking the ability to activate NLRP3 inflammasomes and signal IL-1 and IL-17 did not respond to ICD inducers, such as anthracyclines, a chemotherapy agent.^235^ However, even though together with dying tumor cells that released ATP, an inflammasome inducer, DCs lacking inflammasome activity could not effectively activate CTLs which warrants further investigation.^234^ Other representative DAMPs released by ICD and molecular mechanisms for DAMP-mediated activation of the immune system in TME have been elaborated in detail by Hernández et al., etc.^236^

## Ferroptosis synergizes the antitumor immune response

Similar to other ICDs, ferroptotic cells may release lipid mediators as ‘find me’ signals, which recruit APCs and other immune cells to ferroptotic tumor cells microenvironment.^100^ As well as contributing to the oxygenation of esterified PUFAs as ferroptotic signals, the oxidation products released by ferroptotic cells may also be immunomodulatory.^100^ In response to inducible GPX4 depletion, eicosanoids can be released by cancer cells through ferroptosis.^237^ Nevertheless, with the stimulation of TNF or IL-1β, enhancing GPX4 activity reduces the activation of pro-inflammatory lipid mediators such as LTB4 mediated by NF-κB signals.^238^ It’s noticed that LTB4 is one of the most established pro-inflammatory leukotrienes which plays a key role in carcinogenesis.^239^ With a deeper understanding of free eicosanoids as signaling molecules in modulating immune responses, the interest in esterified eicosanoids biological roles is increasingly growing, which are derived from phospholipids by lipoxygenase (LOXs) activity or from eicosanoids by re-esterification into phospholipids.^240^ In addition, the biological effects of extracellular oxidized PEs or their degradation by oxidation or hydrolysis have been much less uncovered, but it has been demonstrated that lyso-phospholipids can promote APCs to induce apoptosis.^241^ It has also been shown that oxidative state of externalized phospholipids can increase macrophage activity in engulfing and clearing apoptotic cells and macrophages are more likely to phagocytize apoptotic cells whose outer plasma membranes carry peroxidized phosphatidylserine (PS) than cells lacking PS.^242^ Ferroptotic cells secrete an oxidized PL, 1-steaoryl-2-15-HpETE-sn-glycero-3-phosphatidylethanolamine (SAPE-OOH), which is an important “eat-me” signal activating macrophages to phagocytose.^243^ In principle, oxidized PL from ferroptotic cancer cells may modulate immune cells’ activity and response, but this claim has not been experimentally tested.

Some immunosuppressive cells, such as M2-type macrophages, Treg cells, and MDSCs antagonize ferroptosis by high expression of GPX4 or other components to maintain cells activation. Induction of ferroptosis in these cells may cause cell death and reversal of their tumor-promoting function. M1 cells are highly resistant to ferroptosis compared to M2 cells even in the absence of GPX4.^244^ Mechanically, M1 cells express high levels of nitric oxide synthase (iNOS) and produce more NO radicals (NO•) which are inhibited in M2 cells. NO• could react with lipid radicals or lipid peroxidation reactions intermediates, thus replacing GPX4 as a negative regulator of ferroptosis. Thus, in the presence of ferroptosis inducers, M2 cells can undergo ferroptosis or repolarize to the M1 phenotype and subsequently exert antitumor effects.^244^ Similarly, in Tregs activated by TCR/CD28 co-stimulation, GPX4 expression is promoted thereby reducing the occurrence of ferroptosis.^245^ The deletion of the GPX4 gene can lead to excessive accumulation of lipid peroxides (LPOs) and ferroptosis which promote IL-1β production to enhance T helper 17 (Th17) cell antitumor immune response.^246^ Likewise, the function of MDSCs is closely related to lipid transport and metabolism. Polymorphonuclear myeloid-derived suppressor cells (PMN-MDSCs) can depend on myeloperoxidase to undergo lipid peroxidation and transfer lipids to DC cells, blocking the cross-presentation of DC cells and thus exerting tumor-promoting activity.^247^ In addition, MDSCs selectively accumulate arachidonic acid esterified triglycerides (AA-tags), oxidized AA-tags, and prostaglandin E2 (PGE2) but not PL-PUFA and associated lipid peroxides(LPOs) which lead to ferroptosis, thus forming ferroptosis resistance.^248^ What’s more, MDSCs with high expression of the Xc- system consume extracellular cyst(e)ine, but do not transport cysteine to the microenvironment due to the lack of ASC transporter proteins, thereby depriving of the cyst(e)ine required for T cells activation.^249^ Notably, the process of ferroptosis occurring in MDSCs is regulated by the p53 pathway. When p53 protein stability is increased, the production of ROS is inhibited, thereby suppressing ferroptosis in MDSCs.^250^

Immune cells exert antitumor immune functions by releasing cytokines that promote ferroptosis activity in tumor cells. For example, IFNγ released by CTLs activates the Janus tyrosine kinase (JAK) signal and signal transducer and activator of transcription 1 (STAT1) pathway, which downregulates the Xc-system expression and increases intracellular stored iron content thereby inducing ferroptosis.^30^ Similarly, transforming growth factor-β (TGF-β1) released by macrophages can inhibit the Xc-system transcription via SMAD signaling thereby promoting ferroptosis.^251^

## Necroptosis synergizes the antitumor immune response

As we know, necroptosis is a form of ICD due to the release of DAMPs. However, effectors in necroptosis such as RIPK1 and RIPK3 can directly regulate the function of immune cells independently of cell death.^252,253^ In support of this, RIPK3-mediated phosphatase phosphoglycerate mutase 5 (PGAM5) activation promotes natural killer T cells-mediated antitumor immune responses by nuclear translocation of nuclear factor of activated T-cells (NFAT) and dephosphorylation of dynamin-related protein 1 (Drp1) in a process independent of the necroptotic pathway.^254^ Necroptotic tumor cells activated by RIPK3 were injected into pre-existing tumors to enhance antitumor immunity in syngeneic melanoma and lung adenocarcinoma models.^255^ The RIPK3 knockout model of lung carcinoma and lymphoma reduced the efficacy of chemotherapy in vivo, which was linked to decreased CD8+ T cell infiltration.^256^ Similarly, RIPK3(−/−) mice exhibit NF-κB inactivation and impaired secretion of cytokines IL-1β, IL-23, and IL-22, which in turn lead to DC cell dysfunction in damaging inflammation and tissue repair.^257^ It is revealed RIPK3 plays an important role in NF-κB activation, expression of innate inflammatory cytokines, and involvement in tissue repair of DC cells.^257^ Likewise, another study demonstrated the presence of inflammatory gene expression indepentent of plasma membrane rupture caused by necroptosis.^258^ Forced dimerization of MLKL induced-necroptosis promotes inflammatory cytokine release at much lower levels than that of necroptosis induced with TNFα-RIPK-MLKL-NF-κB pathway, suggesting that cell-autonomous inflammatory cytokine expression synergizes with DAMPs release to mount an immune response.^259,260^

Combined with the above, two strategies are currently available to trigger antitumor immunity through necroptosis.^40^ In 2016, Aes et al. first demonstrated that necroptosis of tumor cells is one of the ICD, through which necroptotic cells can release DAMPs to DC cells to trigger antigen presentation and thus activate cytotoxic CD8+ T lymphocytes.^151,261,262^ Furthermore, unlike the study by Aes et al., another study found that fibroblasts in the tumor microenvironment(TME) through necroptosis induced the robust immune response via NF-κB signaling rather than MLKL-mediated cytolytic-dependent DAMPs release.^255^ In mice tumor model in which DAMPs receptor expression is absent, fibroblasts undergoing necroptosis still inhibited tumor growth.^255^ Similarly, Yatim et al. also emphasized the necessity of NF-κB for initiating the immune response and the interaction between necroptosis and TME. During necroptosis, inflammatory mediators released from dead cells are not sufficient to initiate CD8+ T cells, whereas separating NF-κB signaling from necroptosis decreases the efficiency of immune response initiation.^263^

Interestingly, in contrast to the above, high expression levels of RIPK1 and RIPK3 in human pancreatic cancer cells predicted enhanced migration and invasion of tumor cells,^41^ whereas low expression of MLKL was linked to reduced overall survival (OS) in patients with early resectable pancreatic cancer and reduced recurrence-free survival and OS in pancreatic cancer patients receiving adjuvant chemotherapy,^264^ which suggests necroptosis effectors differentially influence tumor pathogenesis in different contexts, and this heterogeneity has not been explained so far. Furthermore, in some cases, RIPK1 is not essential for tumor development. For example, researchers have found that RIPK1 inhibitors do not suppress tumor growth in genetically engineered mice models of pancreatic cancer.^265^ And it has been shown that in mouse mammary tumors, knockout of ZBP1 and MLKL, but not RIPK1, reduces lung metastasis.^266^ We may therefore conclude that RIPK1 regulates tumor growth through its scaffolding function rather than its kinase activity.^260^

The role of cytokines and DAMPs such as HMGB1, ATP, etc. has been described in detail in the pyroptosis section of this paper. In addition to the factors secreted directly by cells undergoing ICD, the immune response is further amplified by the activity of APCs. The role of APCs in the clearance of dying cells is dependent on the mode of death, leading to either anti-inflammatory responses (when apoptotic cells are cleared) or pro-inflammatory responses (when pyroptotic or necroptotic cells are cleared). To be specific, when apoptotic cells are engulfed, phagocytes induce secretion of anti-inflammatory factors such as IL-10 and TGF-β and inhibit the release of pro-inflammatory cytokines and chemokines such as IL-6, IL-1β, CCL2, CCL3.^267,268^ In contrast, as a result of phagocytosis of necroptotic colon carcinoma cells, DCs mature and cross-present to CD8+ T cells, promoting the activity of CD8+ T cells and the production of IFNγ.^261^ Thus, the release of inflammatory mediators directly from the cells through ICD along with DCs maturation and CD8+ T cells activation causes a strong immune response.^269^ We can easily draw a conclusion that pyroptosis and necroptosis are able to induce inflammation through the release of DAMPs and cytokines as well as the change of APCs responsible for phagocytosing dying cells.

## Autophagy, pyroptosis, ferroptosis, and necroptosis antagonize the antitumor immune response

Although autophagy, pyroptosis, ferroptosis, or necroptosis are generally considered to contribute to the immune response against tumors, studies have concluded that the survival, proliferation, differentiation, and activation of immunosuppressive cells including Treg cells, M2 macrophages, MDSCs, etc. are also dependent on these RCDs under certain circumstances. Moreover, immune-promoting cells are negatively regulated by several RCDs. In addition, the release of DAMPs during ICD promotes the development of inflammatory responses favoring tumor growth in addition to stimulating antitumor immune responses (Figs. 2, 3, and 4).

## Autophagy antagonizes the antitumor immune response

Autophagy may enhance or inhibit the growth, development, and functional performance of immune cells, depending on whether they have tumor-promoting or antitumor function.^159,270,271^ In addition, autophagy is also involved in antigen-presentation component of the adaptive immune response.^42^ Overall, autophagy facilitates tumor cell evasion from immune surveillance, leading to intrinsic resistance to antitumor immunotherapy.

Autophagy is required by Treg cells to suppress antitumor immune responses. For example, human melanoma-infiltrating Treg cells express high levels of arginase 2 (ARG2), leading to intracellular arginine degradation and inhibiting activation of arginine-mediated mTOR, which may activate autophagy.^272^ In autophagy-deficient Treg cells, enhanced glycolytic activity and loss of the characteristic forkhead box protein P3 (FOXP3) expression are induced through the mTOR-MYC pathway thereby increasing apoptosis.^273,274^ Consistently, silencing of key molecules involved in autophagy including Beclin1, ATG5, and PI3 kinase class III (PI3K3) may lead to impaired function of Treg cells.^275,276^ In the TME, IL-6 and CCL2 trigger autophagy by binding to interleukin 6 receptor (IL-6R) and CC chemokine receptor 2 (CCR2), respectively, which is essential for macrophage polarization to the immunosuppressive M2 phenotype.^277,278^ Furthermore, it was recently shown that HCC-acquired TLR2 signaling inhibited the NF-κB signaling pathway and thus derived macrophage polarization toward M2 phenotype, which was achieved by the selective autophagy-mediated degradation of NF-κB p65.^279^ Inhibition of autophagy can restore NF-κB activity and induce high levels of M1-like cytokine production by M2-polarized macrophages. In addition, TLR2 signaling can promote sustained phosphorylation of MAPK1 and MAPK3, which stimulates autophagy-dependent NF-κB regulation.^279^ However, autophagy may be inhibited in M2 macrophages through LPS or bacterial infection, which is regulated by mTOR pathway activated by TLR4.^280^ MDSCs are also supported by autophagy for survival and development. Glycolysis reduces partial hepatic enrichment activating protein expression in triple-negative breast cancer by blocking AMPK-ULK1 signaling and autophagy formation, which reduces granulocyte colony-stimulating factor (G-CSF) and granulocyte-macrophage colony-stimulating factor (GM-CSF) expression, thus preventing MDSC developments.^281^ MDSCs, in turn, activate AMPK, stimulate autophagy, and promote the expression of anti-apoptotic factors MCL-1 and BCL-2, thereby promoting multiple myeloma development.^282^

Autophagy helps tumors evade surveillance of CTLs, thereby developing immune tolerance. For example, autophagy induced by 5- hydroxytryptamine/5-hydroxytryptamine 1a receptor (5HT/5-HT1aR) signaling pathway facilities an immunosuppressive NSCLC environment and tumor cell resistance to CTLs-mediated lysis through STAT3 phosphorylation.^198^ Further, autophagy-deficient host mice have tumor-rejecting T cells that are more active than those with adequate autophagy.^256^ Compared with autophagy-proficient tumor models, the augmented infiltration of immune cells and gene expression signatures of activated type I/II IFN pathway can be found in autophagy-deficient tumor models which can be explained by STING activation. In addition, inactivating both Sting and ATG7 gene led to the restoration of tumor growth in mice, showing that tumors are inhibited by innate immunity activation via STING by autophagy impairment.^256^ In addition to type I /II IFN, interestingly, gene expression profiling of tumor models showed that CTLs produced higher levels of IFNγ specifically in autophagy-deficient tumor models. IFNγ gene (IFNG) is also involved in antigen presentation and tumor suppression on autophagy-deficient hosts.^256^ Besides, the IFNG and ATG7 gene defect restored defective growth of tumors, showing the killing effects on tumor of IFNG induced by loss of host autophagy. For instance, the immunosuppressive TME of the liver and immune evasion is attributed to autophagy activation which suppresses innate immune response and thereby antitumor activity of T cells. T cells and IFNG are both required to induce tumor rejection by specific deletion of autophagy in liver hepatocytes.^256^ In order to block selectively autophagy to overcome hepatic autophagy immune tolerance, we need to uncover the exact mechanism by which autophagy loss activates STING-type I/II IFN pathway and IFNG/IFNγ activation in the hepatocytes.

In B16-F10 and 4T1 mouse tumor models, autophagy of tumor cells induced by hypoxia degrades NK cell-derived GZMB thereby impairing the tumor lysis function of NK cells.^283^ Mechanically, hypoxia-inducible factor-2α (HIF-2α) transports into the nucleus and activates the autophagy sensor inositol 1,4,5-trisphosphate receptor type 1 (ITPR1) to degrade GZMB. Similarly, hypoxia negatively affects DC cells function which is associated with HIF-1α accumulation and DC cells’ autophagy/apoptosis regulated by the PI3K/AKT pathway.^284^

Besides, tumor cells can evade immune surveillance through autophagy to degrade MHCI complexes.^198,285^ For example, in pancreatic cancer cells resistant to ICIs, the MHCI complex is re-transported to the lysosome for selective autophagy by ubiquitin-binding receptor NBR1 and degraded, thereby preventing T cells recognition.^286^ By contrast, inhibition of autophagy restores the level of MHCI complex and improves antigen presentation, enhancing antitumor T cells responses and therefore reducing tumor growth. Similarly, in the presence of ATG5 and ATG7, as a result of endocytosis and autophagic degradation of the MHCI complex in DCs, antigen presentation and CD8+ T cells priming are inhibited, which is reversed in DCs with absence of autophagy.^287^ Recently, one study shows that radiotherapy-induced autophagy increases CD8+ T cells infiltration by modulating MHCI expression in NSCLC, but the direct relationship of MCHI expression with autophagy is still unclear.^288^ Likewise, E3 ubiquitin ligase leads to MHCII complex degradation in MDSCs, causing tumor immunity evasion. Conversely, ATG5 deficiency restores MHCII expression on MDSC surfaces.^289^ Results from these studies suggest that tumor immune escape might be facilitated by autophagic degradation of MHCI/II complex in both cancer cells and immune cells.

## Pyroptosis antagonizes the antitumor immune response

It is noticed that as effector molecules during pyroptosis, whether cytokines play a synergistic or antagonistic role in antitumor immunity depends on the tumor microenvironment. During pyroptosis, activation of inflammasomes can promote the maturation and release of inflammatory factors such as IL-18, IL-1β, IL-10, etc. which may inhibit antitumor immune effects or cause an inflammatory cascade response, thereby promoting tumor development under particular circumstances. Expression or secretion level of IL-18 is detected in different cancer cells in comparison with normal tissue.^290^ For example, in lymphoma, NLRP3 inflammasome-induced IL-18 contributes to promoting proliferation, inhibiting apoptosis in cancer cells and reducing drug resistance by interfering with the balance of c-Myc/TP53 protein and Bcl-2/Bcl-2 associated with Bax protein.^291^ Similarly, the inflammatory adapter ASC/IL-18 signaling pathway has a tumor-promoting effect in gastric cancer. Further analysis revealed that the specific effect of IL-18 was associated with high expression of IL-18 gene in gastric cancer epithelial cells, whereas IL-1β was preferentially expressed in immune cells whose knockdown did not inhibit gastric carcinogenesis.^292^ And IL-18 induces migration of breast cancer cells through downregulation of claudin-12 as well as activation of the p38-MAPK pathway.^191^ Besides, IL-18 is able to induce angiogenesis which leads to increased migration/invasion in tumors and immune escape.^293,294^

Aside from IL-18, in cancer patients and experimental tumor models, raised levels of IL-1β are also associated with a worse prognosis, carcinogenesis, and cancer invasion.^295,296^ For instance, IL-1β mediates the proliferation and invasion of colon epithelial cancer cells through the stromal cyclooxygenase-2 (COX-2) signaling pathway.^297^ In prostate cancer, IL-1β promotes cancer cells proliferation and metastasis by activating MAPK-mediated IL-8 production.^298^ By maintaining a microenvironment for cancer stem cells and promoting angiogenesis, IL-1β contributes to tumor growth and progression. For example, dependently expressing vascular endothelial growth factor (VEGF) by IL-1β can pave the way for metastasis and modulate adaptive immune response.^299,300^ Notably, in a pancreatic cancer model, triggered by NLRP3 inflammasomes, tumor cell-derived IL-1β is involved in the construction of an immunosuppressive TME in which infiltrating CD8+ cytotoxic T cells are reduced while M2 macrophages, MDSCs, CD1HICD5+ regulatory B cells, and Th17 cells are increased, thus promoting pancreatic tumorigenesis in a xenograft mouse model.^301^ Specifically, macrophages infiltrated in tumors are stimulated by tumor cells and T cell-derived cytokines such as IL-1 to induce a polarized M2 phenotype characterized by IL-4 and IL-10, etc.^302,303^ IL-1β production induces CCL2 expression via inflammasome activation in TAMs and tumor cells as well, thereby governing the recruitment of myeloid cells into tumors, providing an inflammatory microenvironment and promoting breast cancer progression.^304,305^ In addition to TAMs, IL-1β also plays a role in the proliferation and migration of MDSCs regulated by the IL-1RI/NF-κB pathway.^306^ An experiment involving cells that were transfected with an IL-1β expression vector and injected into mice has demonstrated that mice that received transfected 4T1 tumor cells infiltrated with MDSCs at increased levels.^307^ In addition, the correlation between increased levels of serum IL-1β and a greater number of MDSCs and Tregs in peripheral blood reflects the importance of IL-1β as a proliferating factor for MDSCs.^308^ Furthermore, IL-1β upregulated COX-2, which encodes prostaglandins that are responsible for MDSCs expansion.^309^ Tissue-resident endothelial cells are activated by MDSCs to produce VEGF and other angiogenic factors with the stimulation of IL-1β and other pro-inflammatory agents.^310^ Aside from direct effects of IL-1β and its target genes on MDSCs, elevated CCL2 induced by IL-1β within TME promoted the recruitment of MDSCs.^304^

IL-1β has already been implicated in the transition from chronic inflammation to tumor development. It has been established that various tumors, including colorectal cancer, gastric cancer, liver cancer, lung cancer, and bladder cancer, can be triggered by persistent inflammation.^311^ Hepatitis C virus infection, for instance, induces hepatic inflammation that triggers the evolution from fatty liver disorder to fibrogenesis, and finally HCC induced by IL-1β.^312^ Helicobacter pylori (HP) infection, the most typical bacterial infection which is closely related to gastric cancer can also induce the amount of IL-1β production.^313^ The development of gastritis and gastric tumor have been linked to genetic polymorphisms in the IL-1β gene.^314^ An additional mechanism underlying the increased risk of gastric cancer associated with IL-1β is its ability to induce aberrant DNA methylation.^315^ Besides, it seems that IL-1β can influence chronic obstructive pulmonary disease (COPD) airway inflammation by upregulation in small airway epithelial cells of COPD patients.^316^ Although as a known risk factor, specific pathways of lung cancer developed by COPD is poorly known and IL-1β seems to play a profound role in this regard. In addition, 3-methylcholanthrene (3MCA), a chemical carcinogen, induces the development and invasiveness of tumors if it is exposed to IL-1β.^317^ Furthemore, chronic inflammation results in the induction of immune-suppressive MDSCs, TAMs, and NK cells mediated by IL-1β.^318^ To sum up, the key mechanisms of IL-1β-mediated tumor development include infiltration of immunosuppressive cells,^319^ tumor angiogenesis^320^, and driving chronic inflammation.^312,313^

IL-10-induced adaptive immunosuppression is also involved in the development of pancreatic cancer.^321^ IL-33 has also been regarded as a new type of danger signal released from pyroptotic cell that targets various immune cells and boasts anti- or pro-inflammatory properties depending upon the disorder.^322^ In reference to the above mentioned, IL-1β and IL-18, etc. as immunomodulatory cytokines have been attributed to either initiate adaptive antitumor responses or inhibit antitumor immune effects depending on the makeup of cytokine milieu.^269,323^ Furthermore, Liu’s study found that CAR-T cells could promote GSDME-mediated tumor cell pyroptosis by releasing perforin and GZMB in B-lymphocytic leukemia and solid tumor cells. Nevertheless, pyroptosis can be triggered again in macrophages by pyroptosis releasing factors from tumor cells, leading to the release of cytokines such as IL-6 and IL-1β, which in turn triggers cytokine release syndrome (CRS).^324^

## Ferroptosis antagonizes the antitumor immune response

It is intriguing to speculate that a small part of cells undergoing ferroptosis in the TME may inhibit the immune system, which is mediated by the DAMPs such as KRAS-G12D, HMGB1, 8-hydroxyguanosine (8-OHG) through ferroptosis. During ferroptosis, KRAS-G12D may be released by pancreatic cancer cells, whose exocytosis is dependent largely on the ability to form amphisomes by fusing autophagosomes with multivesicular bodies.^325^ KRAS-G12D promotes M1 phenotype polarization to M2 phenotype by binding to advanced glycosylation end product-specific receptor (AGER) and induces adaptive immunosuppression by releasing arginine (ARG), IL-10, and TGF-β thereby stimulating tumor growth.^325^ Likewise, HMGB1 released by iron-addicted cancer cells promotes inflammatory responses in macrophages by binding to AGER.^326^ Furthermore, iron-addicted cancer cells activate STING-dependent DNA sensor pathways in macrophages by releasing 8-OHG in the presence of GPX4 depletion, promoting the release of cytokines such as IL-6 and nitric oxide synthase 2 (NOS2) to form an inflammatory tumor microenvironment that supports pancreatic cancer.^327^

Similarly, it has been demonstrated that ferroptosis in cancer cells is linked to increased expression of post-transcriptional gene silencing (PTGS2) and the release of PGE2.^100^ We may infer, if the sufficient levels are achieved, antitumor immune response will be converted to immunosuppressive responses,^328^ leading to progressive tumor growth, although more related experiments requires to be validated.^329^ It is interesting to note that PGE2 is released far prior to all cell deaths, suggesting that suppressed GPX4 activity may indeed be sufficient to sustain the PTGS2-active state.^100^ PGE2, induced by cancer cells through ferroptosis promotes the recruitment and activation of MDSCs and M2 macrophages and inhibits the antitumor function of NK cells, DCs, and cytotoxic T cells. Mechanically, in myeloid cells, PGE2 can activate DNA methyltransferase 3A (DNMT3A), leading to DNA methylation and suppressing immunogenic gene expression.^330^ A study has shown that PGE2 can exert immunosuppressive effect in cell lines based on a melanoma mouse model engineered to express BrafV600E mutation, the most prevalent mutation in patients. It was found that PGE2 production is sufficient to inhibit DC-dependent antitumor immunity mediated by CD8+ T cells in this model.^331^ Besides, considered as a major immunosuppressive mediator, PGE2 directly suppressed cytotoxic T cells activity, consequently interfering with multiple aspects of anticancer immunity.^332^ PGE2 also compromise DCs directly by reducing the amount of chemokine receptors that induce the recruitment into tumors.^100^ Further research found that PGE2 reduced the amount of DCs infiltrated into TME by suppressing chemokines CCL5 and XCL1.^329^ Although the action of PGE2 and its downstream signaling has not been elaborated more detailedly, current study has provided strong evidence that PGE2 has immunosuppressive effect towards NK cells.^333^

As the stimulation of ferroptosis, ROS acts a vital role in the modulation of immunity in human malignancies in addition to oxidative stress.^334^ The presence of high ROS inhibits T cells activation and proliferation, while low ROS can restore the cytotoxic effects of T cells.^335^ ROS suppress the formation of TCR and MHC antigen complexes in T cells, thus inhibiting immune responses.^335^ What’s more, ROS scavengers is able to improve the CTLs activation by activating superoxide dismutase 2 (SOD2).^336^ In addition, the ability of CAR-T cells to kill cancer cells has been linked to lower levels of intracellular oxidative stress.^337^ Thus, CAR-T cells in combination with catalase (ROS inhibitors) demonstrated better antitumor response even when exposed to oxidative stress on the extracellular surface.^337^ Besides, researchers also observed that CTLs extracted from murine with the treatment of PD-1/L1 antibody might have high mitochondria ROS and elevated O2− microenvironment, which results in compromised CTLs action and inhibited immune response. Thus, Metformin (a ROS inhibitor) combined with PD-1 inhibition enhanced intratumor T cell activation and proliferation, resulting in tumor clearance and alleviating tumor inflammation through the decreased level of tumor hypoxia.^338^ In addition to CTLs, multiple studies have shown that oxidative stress or ROS caused Tregs to suppress the immune system within a tumor niche. The mitochondrial complex III appears to be required for the inhibition of Tregs function.^339^ Kunisada and colleagues also found that metformin reduced the amount of tumor-infiltrating Tregs by suppressing the differentiation of naive CD4+ T cells.^340^ The MDSCs induced by tumors also inhibited T cells proliferation and increased colorectal carcinoma cell growth by producing ROS^341^ while the negative effect of MDSCs can be suppressed by catalase, thus restoring T cells action.^342^ ROS are also involved in the activation of macrophage signaling. It has been demonstrated by Lin X et al. that ROS may stimulate an invasive phenotype in TAMs derived from melanoma through the secretion of TNFα.^343^ Researchers have found several mitochondrial genes highly expressed in TAMs derived from melanomas, indicating ROS is the major cause of oxidative stress within TAMs.^334^ To sum up, the key mechanisms of ROS involved in TME in modulating tumor immunity remain to be unknown, which need more research.

In addition, many immune cells are sensitive to ferroptosis, including CD8+ T cells, NK cells, and DC cells. Stimulation of ferroptosis by inhibition of GPX4 activity can reduce the specific killing function of these immune cells. CD36 expression on the cell surface has been reported to be crucial for fatty acid or oxidized lipid-induced ferroptosis. Significant lipid peroxidation activity can occur in CD36-positive CD8+ T cells, which results in ferroptosis induced by GPX4 inhibitors, leading to reduced release of IFNγ and thus inducing immunosuppression.^344,345^ Although there are no relevant studies directly with ferroptosis in NK and DC cells, Poznanski et al. demonstrated that protein expression associated with ferroptosis, lipid peroxidation, and oxidative damage was increased and had a similar cell morphology to that of cells undergoing ferroptosis in NK cells. Furthermore, oxidative stress associated with lipid peroxidation inhibited glucose metabolism in NK cells leading to their dysfunction.^346^ Similarly, tumor-associated DC cells usually exhibit reduced antigen-presentation capacity due to elevated lipid levels, which is associated with ferroptosis susceptibility.^98,347^ For example, the 12/15-lipoxygenase(12/15-LO) inhibits DCs maturation and activation as well as dampens the differentiation of T helper 17 cells by generating phospholipid oxidation products that induce antioxidant responses dependent on nuclear factor erythroid 2-related factor 2 (NRF2).^348^ Similarly, Ramakrishnan et al. showed that various oxidized lipids in DCs, blocked the cross-presentation of exogenous antigens by reducing the expression of MHCI complexes on cell surface.^349^

## Necroptosis antagonizes the antitumor immune response

Induction of necroptosis can generate an immunosuppressive TME in which tumor growth is allowed. A mouse model of pancreatic cancer showed that knocking out RIPK3 or RIPK1 inhibited oncogenic progression. RIPK3-dependent necroptosis of pancreatic cancer cells results in increased expression of sin3A-associated protein 130 (SAP130) and release of chemokines such as C-x-c motif chemokine ligand 1 (CXCL1) and CXCL5, leading to the recruitment of immunosuppressive cells such as MDSCs to form immunosuppressive TME mediating the migration and invasion of cancer cells.^350,351^ Similarly, RIPK3 signaling in MDSCs increases tumor size by expanding IL17-producing T cells in tumor models.^352^ In addition, RIPK1 expression was found to be upregulated and then STAT1 was inhibited in TAMs which were differentiated into immunosuppressive M2 cells.^353^ Inhibition of RIPK1 results in cytotoxic T cell activation as well as T helper cell differentiation to a mixed Th1/Th17 cell phenotype, stimulating antitumor immunity to suppress ductal adenocarcinoma of pancreas (PDA) growth in mice.^353^ Notably, the tumor-promoting effect of RIPK1 occurring here is independent of the synergistic effect of RIPK3 on it. However, the inhibition of RIPK1 in a mouse model of pancreatic ductal adenocarcinoma, however, did not result in an improvement in overall survival or tumor growth.^265^ Noteworthy, there may be differences in immunogenicity between the two groups based on the differences in pancreatic mouse models. And phase I/II trials (NCT03681951) about GSK3145095, one RIPK1 inhibitor, have been aborted in pancreatic cancer and headed back to the company’s research and development. In conjunction with this withdrawal, Patel et al. published results showing that RIPK1 inhibition did not inhibit cancer growth or metastasis. Moreover, the TNFα-induced systemic inflammatory response syndrome is effectively blocked in vivo by RIPK1 inhibitor PK68. Both melanoma cells and lung cancer cells are repressed by pretreatment with PK68 achieved by reprogramming of intra-tumoral macrophages. Also, conflicting results have been generated in the study on the impact of RIPK1 inhibitor on metastasis, showing GNE684 had no effect as compared to PK68.^265,354^ Therefore, different RIPK1 inhibitors have shown varying results and reinforced a nebulous role of RIPK1 in cancer, thus, require further study. In addition, deletion of MLKL in breast cancer cells reduced lung metastasis.^355^ In some cancer types, tumor cells have also shown a tendency to induce necroptosis in endothelial cells, causing the transendothelial migration and metastasis of tumor cells via expression of amyloid precursor protein.^356^ There is some uncertainty whether the effects on metastasis result from necroptosis itself or disruption of endothelial barriers. However, we can infer that the activation of necrosome enhances cancer progression. A more complex interaction has been observed between different cell types induced necroptosis and early disease outcomes.

Multiple of evidence suggests that DAMPs released from cells through necroptosis may also recruit inflammatory cells and release regulatory cytokines such as IL-1α, IL-18, etc. to trigger inflammation and promote tumor development by promoting angiogenesis, cancer cell proliferation, and thus metastasis.^4,41,357–359^ The role of cytokines and DAMPs such as HMGB1, ATP, etc. has been described in detail in the pyroptosis section of this paper. Interestingly, MLKL signaling activates NLRP3 inflammasomes, thereby activating CASP1 and triggering the release of the pro-inflammatory cytokine IL-1β which suggests that pyroptosis is involved in the pro-inflammatory process of necroptosis.^360,361^ Recently, Gutierrez et al. found that GSDMD required for cell lysis as a substrate for CASP1 during pyroptosis was not necessary for MLKL-dependent necroptosis and IL-1β secretion.^360,361^ We therefore infer that the activation of cytokine release by MLKL may occur before cell lysis, suggesting that MLKL is an endogenous activator of NLRP3 inflammasomes in a GSDMD-independent manner. Activated inflammatory cells may also release reactive nitro intermediates (RNI) and ROS, which promote tumorigenesis by damaging deoxyribonucleic acid and causing genomic instability.^357^ We have detailedly discussed the effects of ROS on immune modulation in the ferroptosis section of this paper.

## New advances in targeting autophagy, pyroptosis, ferroptosis, and necroptosis in immunotherapy

### Anticancer drugs targeting autophagy, pyroptosis, ferroptosis, and necroptosis

Targeting autophagy, pyroptosis, ferroptosis, and necroptosis to develop new anticancer drugs for clinical application is a long process, whereas current studies have shown that many drugs approved for clinical application exert potent antitumor activity by inducing (or inhibiting) these types of non-apoptotic RCD^40^ (Table 2). Currently, the only clinically approved autophagy inhibitors are chloroquine derivatives, which have a long history in the treatment of malaria and rheumatic diseases. To be specific, chloroquine (CQ) and hydroxychloroquine (HCQ) inhibit autophagosome degradation by inhibiting the lysosomal acidification.^362^ These drugs, especially HCQ, have been re-used in many clinical trials for the treatment of various cancers.^363–365^ Metformin inhibits cancer cell proliferation by inducing indirect pyroptosis activation through CASP3.^366^ In detail, metformin causes mitochondrial dysfunction and activates the AMPK/sirtuin1/NF-κB pathway, promoting the accumulation of Bax which results in CASP3 activation and GSDME cleavage. In esophageal squamous cell carcinoma (ESCC), metformin can induce GSDMD-mediated pyroptosis by targeting the miR-497-PELP1 axis.^367^ Sorafenib, an FDA-approved anticancer drug for the treatment of HCC, renal cell carcinoma, and thyroid cancer, as the Xc- system inhibitor, prompts ferroptosis by depleting the antioxidant GSH making the GPX4 system inactive.^368,369^ Nevertheless, some cancers are inevitably resistant to sorafenib, which may be caused by the upregulation of the non-GSH-dependent thioredoxin antioxidant pathway.^370,371^ Conversely, octreotide, which is FDA-approved for ovarian cancer treatment, can directly target and inhibit GPX4 to induce ferroptosis, which greatly overcomes the limitations of conventional drugs.^372^ In addition, the combination of lapatinib, a tyrosine kinase inhibitor used for breast cancer treatment, and siramesine, a lysosomal-disrupting hemolysin drug, can synergistically cause ferroptosis by disrupting iron transport and inducing lipid peroxidation in cancer cells.^373^ Shikonin, a natural naphthoquinone, is the first reported small-molecule drug to prompt necroptosis. It has been found to exert antitumor effects via the RIPK1/RIPK3-dependent necroptosis pathway in various cancers such as pancreatic cancer, and triple-negative breast cancer.^374,375^

**Table 2:** Summary of clinically approved drugs that may target autophagy, pyroptosis, ferroptosis, and necroptosis in cancers and their effects on antitumor immune response

Drug name	Indications	Mechanisms	Effects on tumor cell death	Cancer type	References	Effect on antitumor immune response	References
HCQ/CQ	Malaria and rheumatologic disorders	Deacidify the lysosome and block fusion of autophagosomes with lysosomes	Autophagy inhibition	Glioblastoma/NSCLC/breast cancer/PDA	^363–365^	Enhanced antitumor immune response	^286^
Metformin	Hypoglycemic drug	Target AMPK/SIRT1/NF-κB pathway or miR-497/PELP1 axis	Pyroptosis induction	Esophageal carcinoma	^366,367^	Enhanced antitumor immune response	^454^
Anthocyanin	Nutrient	Enhance the expression of NLRP3, CASP1, and IL-1β	Pyroptosis induction	Oral squamous cell carcinoma	^455^	Enhanced antitumor immune response	^456^
Paclitaxel	Anticancer drug	Activate CASP 1/GSDME	Pyroptosis induction	Lung cancer	^457^	Enhanced antitumor immune response	^458^
BRAF and MEK Inhibitor	Anticancer drug	Promote cleavage of GSDME and release of HMGB1	Pyroptosis induction	Melanoma	^459^	Enhanced antitumor immune response	^459^
Shikonin	Thrombocytopenia drug	Activate RIPK1 and RIPK3	Necroptosis induction	Osteosarcoma/Pancreatic cancer	^374,460^	Enhanced antitumor immune response	^461^
Resibufogenin	Heart failure drug	Induce RIPK3-MLKL-dependent necroptosis/GPX4 inactivation mediated ferroptosis/ROS-NF-κB-CASP1-dependent pyroptosis	Necroptosis /ferroptosis/pyroptosis induction	Colorectal cancer/ Lung cancer	^462,463^	Unknown	–
Sorafenib	Anticancer drug	Inhibit system Xc-function	Ferroptosis induction	Hepatocellular carcinoma	^368^	Enhanced immune suppression	^464^
Lapatinib	Anticancer drug	Increase the expression of transferrin and cytosolic ROS	Ferroptosis induction	Breast cancer	^373^	Unknown	–
Octreotide	Anticancer drug	GPX4 inactivation	Ferroptosis induction	Ovarian cancer	^372^	Enhanced antitumor immune response	^465^
Stains	Hyperlipemia drug	HMGCR inhibition	Ferroptosis induction	Cancer cell	^416^	Enhanced antitumor immune response	^466^
Trigonelline	Nutrient additive	Inhibit the Nrf2-ARE pathway	Ferroptosis induction	Head and neck cancer	^417^	Unknown	–
Artesunate	Antimalarial drug	Decrease cellular GSH levels and increase lipid ROS levels	Ferroptosis induction	Head and neck cancer	^417^	Enhanced antitumor immune response	^467^
Sulfasalazine	Anti-inflammatory drug	Inhibit system Xc-function	Ferroptosis induction	Head and neck cancer	^418^	Unknown	–
Glutamate	Nutrient	Inhibit system Xc-function	Ferroptosis induction	Cancer cell	^70^	Enhanced immune suppression	^468^
5-FU	Anticancer drug	Induce NF-κB/TNF-α/RIP1 dependent necroptosis/ CASP3/GSDME dependent pyroptosis	Necroptosis/pyroptosis induction	Colorectal cancer/Gastric cancer	^469,470^	Enhanced antitumor immune response	^471^
Iron	Nutrient	Induce lipid peroxidation/Tom20-Bax-CASP-GSDME pathway	Ferroptosis/pyroptosis induction	Melanoma	^472,473^	Enhanced antitumor immune response	^474^
Doxorubicin	Anticancer drug	Induce pyroptosis via DFNA5/autophagy via eEF-2K	Pyroptosis/autophagy induction	Melanoma	^475^	Enhanced antitumor immune response	^476^
Cisplatin	Anticancer drug	Activate CASP3/GSDME/GPX4 inactivation	Pyroptosis/Ferroptosis induction	Lung cancer	^457,477^	Enhanced antitumor immune response	^478^

The development of new compounds targeting autophagy, pyroptosis, ferroptosis, and necroptosis is ongoing. Lysosomal drugs based on the CQ structure are in development including Lys05,^376^ DQ661,^377^ and DC661.^378^ It has been shown that these compounds bind then inhibit the lysosomal enzyme palmitoyl protein thioesterase 1 (PPT1) to inhibit autophagy.^379^ In addition, studies on inhibitors of different targets in autophagy including ULK1 inhibitors such as SBI-0206965^380^ and ULK101,^381^ VPS34 inhibitors such as SAR405,^382^ compound 13,^383^ SB02024,^384^ and ATG4B inhibitors including S130^385^ and FMK-9a,^386^ and NSC185058,^387^ all of which have been shown to have excellent antitumor activity. Besides, the autophagy activator adiponectin ADIPOQ and 2-aminonicotinonitrile compound (w09) significantly inhibit breast cancer growth by stimulating autophagy through the serine/threonine kinase 11 (STK11)-AMPK-ULK1 and EGFR-mediated RAS-RAF1-MAP2K-MAPK1/3 pathway, respectively.^388,389^

α-NETA induces pyroptosis in epithelial ovarian cancer cells through the GSDMD/CASP4 pathway.^390^ In colorectal cancer cell lines expressing high levels of GSDME, the combination of TNFα and CHX activates members of the BCL2 family, BAK/BAX, which leads to mitochondrial outer membrane permeability (MOMP) and mediates pyroptosis.^391^ Polyphyllin VI (PPVI) induces pyroptosis in NSCLC via the ROS/NF-κB/NLRP3/GSDMD signaling, thereby inhibiting the proliferation of NSCLC.^392^ Bionanoparticles (BNP) loaded with chemotherapeutic agents have antitumor activity in breast cancer, showing that mitochondrial damage and activation of CASP3 is induced upon photoactivation, which subsequently leads to GSDME-mediated pyroptosis.^393^ Similarly, aurora kinase A (AURKA) inhibitors exert an inhibitory effect on pancreatic cancer growth by inhibiting the activity of AURKA to initiate the assembly and activation of necrosome thereby inducing necroptosis.^394^ The death receptor ligand TNF-related apoptosis-inducing ligand (TRAIL) acts on TNFR1, mediated by RIPK1/RIPK3 to lead to necroptosis, which is notably regulated by ROS as well as related caspases.^395^ CD95 ligand (CD95L) binds to its receptor CD95 and induces necroptosis by downregulating cIAPs.^396^ In addition, selenium nanoparticles induce ROS-mediated necroptosis in prostate cancer cell lines, whereas this cell line only relies on RIPK1 and does not require the activation of RIPK3 and MLKL.^397^ Ag/Au bimetallic nanoparticles trigger mixed programmed cell death including necroptosis and pyroptosis in p53 deficient cells while they also trigger the release of IL-1β and HMGB1.^398^

Besides, zero-valent-iron nanoparticle (ZVI-NP) in preclinical models could lead to mitochondrial dysfunctions, oxidative stress as well as lipid peroxidation, inducing ferroptosis in lung carcinoma cell lines.^399^ Ferroptosis inducers, BSO affects GSH synthesis, rendering GPX4 inactive, which in turn reduces tumor burden in mice^371^ and increases the sensitivity of melanoma and neuroblastoma cells to chemotherapy.^400,401^ Similarly, cyst(e)inase, a compound that increases the efficiency of GSH consumption and thus inhibits GPX4 activity, suppresses the growth of prostate and breast cancer xenografts and increases the survival of mice with chronic lymphocytic leukemia models.^402^ Likewise, drugs loaded GSH-bioimprinted nanocomposites are engineered to enhance anti-leukemogenesis by depleting GSH and disrupting intracellular redox status, thus inducing ferroptosis.^403^ In addition to inhibiting GPX4 by affecting GSH synthesis, consumption and depletion, withaferin A increases lipid peroxidation through heme oxygenase 1-mediated heme degradation, thereby inducing ferroptosis in neuroblastoma.^404^

### Autophagy, pyroptosis, ferroptosis, and necroptosis in immunotherapy

**Fig. 5:**
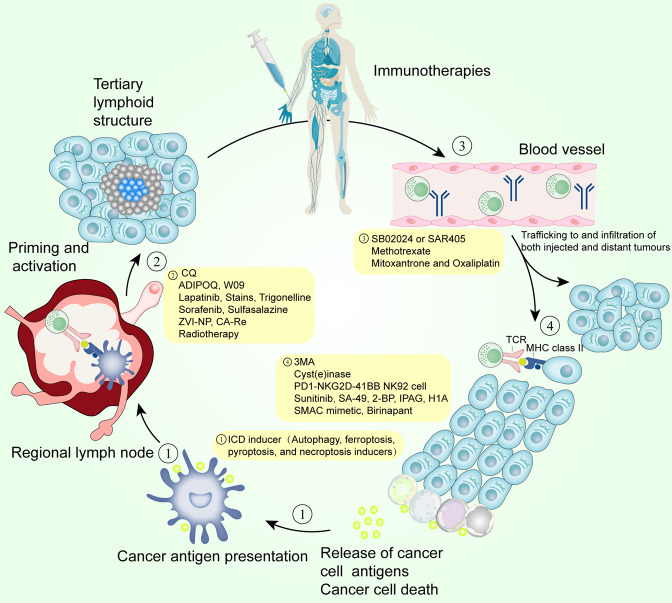
Targeting autophagy, ferroptosis, pyroptosis, and necroptosis for tumor immunotherapy in cancer immunity cycle. Five key processes of cancer immunity cycle are noted in the figure. Agents that target autophagy, ferroptosis, pyroptosis, and necroptosis act synergistically with antitumor immunotherapy on different step of cancer immunity cycle (Autophagy inhibitor: CQ, SB02024, SAR405, 3MA; Autophagy inducer: ADIPOQ, W09, Mitoxantrone, Oxaliplatin, Sunitinib, SA-49, 2-BP, IPAG, H1A; Ferroptosis inducer: Lapatinib, Stains, Trigonelline, Sorafenib, Sulfasalazine, Cyst(e)inase, ZVI-NP; Pyroptosis inducer: Radiotherapy, Methotrexate, PD1-NKG2D-41BB NK92 cell, CA-Re; Necroptosis inducer: SMAC mimetic, Birinapant)

Nowadays, ICIs, especially those targeting PD-1 and PD-L1, have been approved for the treatment of various cancers.^23–25,405^ Great success has been achieved in some solid tumors such as lung cancer and melanoma.^405,406^ Nevertheless, ICIs are limited by the fact that only about one-third of patients respond to ICIs. One of the main factors contributing to primary resistance to ICIs is the lack of tumor-infiltrating T cells, which is one of the characteristics of “cold” tumors whose TME is infiltrated with various immunosuppressive cells such as stromal cells, M2 macrophages, MDSCs, and Treg cells.^407,408^ ICIs may be most effective when in combination with therapies that increase the amount of CD8+ T cells for the treatment of cold tumors. Therefore, we hypothesize that there may be strong potential for the combination of ICIs with ICD induction or inhibitors. In the following, we will address the interaction of targeting autophagy, pyroptosis, ferroptosis, and necroptosis therapy with ICIs (Fig. 5).

As mentioned previously, autophagy is involved in the survival, activation, and functional performance of immune cells.^157,175^ However, in preclinical models of melanoma and breast cancer, the autophagy inhibitor, CQ, did not impair T cells function, suggesting that the immune system may be tolerant to autophagy inhibitors in a certain intensity.^409^ Given the fact that autophagy may antagonize antitumor immune response, autophagy inhibitors may enhance the effectiveness of immunotherapy and overcome resistance to immunotherapy.^410^ For example, CQ blocks autophagy-mediated degradation of MHCI complexes, which together with dual ICIs treatment (anti-PD1 and anti-CTLA4 antibodies) has a synergistic effect and leads to an enhanced antitumor immune response in a mouse model of pancreatic cancer.^286^ Vps34 inhibitor SB02024 or SAR405 results in elevated levels of CCL5, IFNγ, and other chemokines in TME, causing high levels of NK cells and T cells infiltration in myeloma and colorectal cancer models.^411^ Furthermore, in an osteosarcoma model, tumor immunogenicity promoted by photodynamic therapy can be counteracted by ROS-induced autophagy, which can be enhanced by the autophagy inhibitor 3MA by suppressing PD-L1 expression, thereby enhancing the efficacy of photodynamic therapy.^412^ In addition to autophagy inhibitors, induction of autophagy may also enhance the efficacy of ICIs. It has been shown that in human melanoma, expression of the key autophagosomal component LC3-β and other autophagy activators reduces melanoma antigen-A (MAGE-A) protein levels and suppresses the MAGE-Tripartite Motif Containing 28 (TRIM28) complex which predicts resistance uniquely to blockade of CTLA-4, suggesting exploitation of autophagy inducer such as ADIPOQ, w09 for potential therapeutic synergy with CTLA-4 inhibitors.^388,389,413^

Ferroptosis can limit the function of immunosuppressive cells such as TAMs and Treg cells in cold tumors, transforming immunosuppressive TME into an inflammatory TME rich in antitumor immune cells.^244,246,414,415^ Therefore, ferroptosis inducer including Lapatinib, Stains, Trigonelline, etc. may help reverse primary resistance to immunotherapy and enhance the efficacy of ICIs.^373,416,417^ Nevertheless, in tumors with high levels of MDSCs infiltration, ferroptosis inducers may not be a good choice, stemming from the resistance of MDSCs to ferroptosis.^249,250^ MDSCs compete with immune cells for cyst(e)ine, thus depriving T cells of cyst(e)ine and inhibiting T cells activation due to the high expression level of system Xc- but the absence of ASC transporter. From this perspective, ferroptosis inducers targeting system Xc- such as Sorafenib and Sulfasalazine may alleviate MDSC-mediated deprivation of cyst(e)ine, thereby promoting T cells survival and restoring antitumor immune response.^368,369,418^ In preclinical models, in combination GPX4 inhibitor, cyst(e)inase with immunotherapy can synergistically improve T-cell-induced antitumor immunity and triggers cancer cells undergoing ferroptosis.^419^ Moreover, ferroptosis inducer, ZVI-NP also has a key role in augmenting antitumor immune response through polarizing M2 macrophages to M1, reducing the amount of Tregs, lowering the expression of PD-L1 on tumor cells as well as PD-1/CTLA4 on CD8+ T cells, thus maximizing the antitumor effects.^399^ In addition, ferroptosis inhibitor ferrostatin-1 prevents ferroptosis of CD8+ T cells by targeting fatty acids mediated by CD36 and inhibiting lipid peroxidation, exhibits elevated cytokine production and enhances tumor eradication.^344^ More importantly, combined with anti-PD-1 antibodies, ferrostatin-1 has greater antitumor effectiveness.

As pyroptosis enhances the tumor-killing activity of immune cells, it may improve the efficacy of ICIs as the killing mechanism of cytotoxic lymphocytes.^114^ Wang’s study found that ICIs were effective in killing cold tumor cells only in the presence of pyroptosis. Similarly, pyroptosis induction alone did not trigger effective tumor suppression, emphasizing the importance of pyroptosis inducers in combination with ICIs for the treatment of cold tumors.^29^ Lu et al. designed NK92 cells containing a chimeric co-stimulatory transforming receptor (CCCR) that converted inhibitory PD-1 signals into activation signals, effectively enhancing its activity against H1299 lung cancer cells and significantly inhibiting tumor growth in vivo.^420^ Further analysis concluded that it was achieved through GSDME-mediated pyroptosis. A carbonic anhydrase IX (CAIX)-anchored rhenium(I) photosensitizer (CA-Re) shows favorable efficacy in photodynamic treatment for effectively stimulating tumor immunogenicity under hypoxic conditions through GSDMD-independent pyroptosis.^421^ Moreover, the maturation and antigen-presentation capacity of DC cells as well as the activation of CTLs is enhanced by CA-Re, thus killing tumors. Likewise, Zhang et al. designed the engineering covalent organic frameworks (COFs) that could induce durable antitumor immunity through robust induction of GSDME-mediated pyroptosis and the remodeling of TME, thus improving immunotherapy response and restraining tumor metastasis and relapse.^421^ However, induction of pyroptosis may not benefit all immunotherapies. Recent studies have shown that CAR-T cells can quickly causes pyrolysis in targeted tumor cells via GZMB/GSDME/CASP3 pathway. Then, CASP1 is activated via pyroptosis releasing factor, which cleaves GSDMD in macrophages, causing the release of cytokine and cytokine release syndrome (CRS), a serious side event characterized by fever, hypotension, and respiratory failure.^422^ Thus, the development of more efficient drugs causing tumor-specific pyrolysis and decreasing pyrolysis in normal tissues is in urgent need.

Necroptosis inducer can synergize with ICIs in an antitumor context. SMAC mimetics bind and degrade cIAPs, inducing necroptosis to promote antitumor immune responses. In melanoma, the SMAC mimetic, Birinapant sensitizes tumor cells to TNFα-mediated T cells killing and directly regulates immune cell function including B cells, myeloid-derived cells, and cytotoxic lymphocytes by modulating the NF-κB signaling pathway, thus improving the response to ICIs.^423,424^ Similarly, in a mice tumor model, RIPK1-dependent cell death through SMAC mimetics improves the survival benefit of immune checkpoint blockade by activating CD8+ T cells and NK cells.^425^ These data are further supported by studies in glioblastoma, where SMAC mimetics can exert synergistic effects when combined with ICIs or innate immune stimulants to produce durable cures in which single-agent therapy is ineffective.^426^ Moreover, SMAC mimetics has also been suggested to enhance the efficacy of CAR-T cells in the treatment of acute lymphoblastic leukemia in term of mechanism that death receptor signaling is a key mediator of CAR-T cells cytotoxicity.^427^ Vaccinia virus (VACV) is a novel type of immuno-oncolytic therapy based on the mechanism that it can selectively replicate in cancer cells and trigger danger signaling thus augmenting antitumor immunity.^428^ Especially, in syngeneic mouse models, delivering MLKL into tumor cells through VACV causes necroptosis and boosts antitumor immune response directly against neo-epitopes.^428^ Moreover, RIPK3-mediated necroptosis can downregulate the expression of immunosuppressive BACH2/GATA-3 by suppressing KRAS-loaded exosomes, which can potentiate the cytolytic effects targeting tumor cells.^429^

### RCD in chemotherapy, radiotherapy, and targeted therapy

In addition to cold tumors infiltrated by immunosuppressive cells, immune desert tumors are also less responsive to immunotherapy due to their low immunogenicity and lack of immune cell infiltration, therefore, radiotherapy, chemotherapy, and targeted agents may be more effective.^430^ Some targeted therapy (e.g., sorafenib), chemotherapy (e.g., paclitaxel), and radiotherapy may increase the immunogenicity of tumor cells and promote immune cell infiltrated within TME through autophagy, pyroptosis, ferroptosis, and necroptosis, thus enhancing the efficacy of ICIs. In clinical practice, targeted therapy, radiotherapy, and chemotherapy combined with immunotherapy have been applied successfully in various tumors.^431–433^ Therefore, we summarized clinical trials of some novel autophagy, pyroptosis, ferroptosis, and necroptosis inducers/inhibitors, chemotherapy, radiotherapy, and targeted therapy combined with immunotherapy (Table 3).

**Table 3:** Summary of clinical trials testing combinations of immunotherapy with drugs that may target autophagy, pyroptosis, ferroptosis, and necroptosis

Tumor type	Clinical trial phase	Treatment	Potential non-apoptotic cell death pathway	Clinical trial identification number
Advanced NSCLC	III	Pembrolizumab + pemetrexed plus platinum	Ferroptosis/pyroptosis/necroptosis	NCT02578680
Advanced NSCLC	III	Atezolizumab + bevacizumab plus carboplatin plus paclitaxel	Ferroptosis/pyroptosis/necroptosis	NCT02366143
Locally advanced or metastatic urothelial carcinoma	III	Atezolizumab + platinum	Ferroptosis/pyroptosis/necroptosis	NCT02807636
Advanced NSCLC	II	Pembrolizumab + stereotactic body radiotherapy	Ferroptosis/necroptosis	NCT02492568
Metastatic lesions in the liver or lung	I	Ipilimumab + stereotactic ablative radiotherapy	Ferroptosis/necroptosis	NCT02239900
Metastatic renal cell carcinoma	I/II	HCQ + aldesleukin (IL-2)	Autophagy	NCT01550367
Metastatic melanoma	I/II	HCQ + nivolumab/ipilimumab	Autophagy	NCT04464759
Gastrointestinal cancer	I/II	HCQ + cobimetinib + atezolizumab	Autophagy	NCT04214418
Pancreatic cancer	II	HCQ + gemcitabine + nab-paclitaxel + avelumab	Autophagy	NCT03344172
Advanced solid malignancies	I/II	SMAC mimetic + nivolumab	Necroptosis	NCT04122625
Advanced solid malignancies	I	SMAC mimetic + avelumab	Necroptosis	NCT03270176
Pancreatic cancer and colorectal adenocarcinoma	I	SMAC mimetic + pembrolizumab	Necroptosis	NCT03871959

Standard chemotherapy has been considered to inhibit tumor growth mainly through apoptosis.^5^ However, studies have further revealed that chemotherapy can trigger non-apoptotic RCD through calreticulin exposure, autophagic ATP release, and HMGB1 upregulation, leading to increased immune cell infiltration.^434–436^ Gao et al. found that in cholangiocarcinoma cells, methotrexate-induced GSDME-mediated pyroptosis led to activation of tumor-derived macrophages and recruitment of neutrophils at the tumor site to exert antitumor effects.^437^ Decitabine in combination with chemotherapy nanodrugs in cancer treatment can elicit pyroptosis through epigenetics, thereby enhancing the immunological effects of chemotherapy.^438^ In the presence of mitoxantrone and oxaliplatin, autophagy can be activated in mice with CT26 colon tumors, inducing infiltration of DC cells and T cells in TME.^164^ In addition, autophagy in CT26 cells facilitates the secretion of ATP during mitoxantrone treatment.^164^ Extracellular ATP can lead to NLRP3-mediated inflammasome activation, subsequently, recruit APCs into TME to produce IL-1β and activate the antitumor adaptive immune response.^234^ Notably, some chemotherapy agents lead to unfolded protein responses by inducing endoplasmic reticulum stress, which promotes the release of DAMPs to cause ICD.^38^ Compared with oxaliplatin, cisplatin is not effective in inducing autophagy in prostate cancer cells which may be related to invalid unfolded protein response, thus weakens a robust immune response.^439^

The direct effect of radiotherapy on cancer cells is DNA damage, leading to cell cycle arrest or apoptosis.^440^ However, it was found that in some solid tumors, DNA fragments generated by ionizing radiation can be recognized by intracellular DNA sensors such as AIM2 or ZBP1 thereby activating inflammatory signaling and inducing pyroptosis.^441,442^ Cytosolic DNA can also activate another DNA sensor, the STING pathway, providing additional immune stimulation through the production of type I interferon.^443^ Together with inducing pyroptosis, in tumor models, radiotherapy-activated DNA damage response associated kinases such as ataxia-telangiectasia mutated proteins (ATM) inhibit Xc- system leading to reduced cyst(e)ine uptake which enhances tumor lipid oxidation and induce ferroptosis.^28^ This acts synergistically with interferons released by immunotherapy-activated CD8+ T cells, further demonstrating the robust antitumor effects of immunotherapy combined with radiotherapy. Besides, the release of calreticulin and ATP from colon tumors cells through autophagy activated by radiotherapy promotes phagocytosis of tumor cells by DC cells.^444,445^ Stereotactic body radiation therapy (SBRT) combined with oncolytic virus enhances antitumor immunity by altering the M1/M2 ratio of macrophages through necroptosis.^446^ Targeted therapies can also promote ICD and produce immunogenic effects while inhibiting tumor proliferation. Inhibition of cell cycle protein-dependent kinases in a mouse model promotes the release of DAMPs thereby improving antitumor immune response and increasing response to ICIs in mice.^447^ In addition, it is worth noting that many of the clinical kinase inhibitors such as Bcr-Abl inhibitor also target RIPK1 and RIPK3 to improve the efficacy of current cancer-targeted therapies.^448^ MEK inhibitor in combination with autophagy inhibitor can activate TAMs toward an immunogenic M1-like phenotype through STING/type I interferon pathway, which is an attractive therapeutic approach for PDA immunotherapy development.^449^ During a phase III trial of patients with metastatic breast cancer, the receptor protein tyrosine kinase inhibitor sunitinib enhanced the efficacy of ICIs by inducing p62-mediated selective autophagy to downregulate tumor PD-L1 expression.^450^ Likewise, SA-49 treatment facilitates PKCα/GSK3β/MITF-mediated PD-L1 autophagic degradation,^451^ and DHHC3 inhibitor 2-bromopalmitate (2-BP) induces PD-L1 autophagic degradation by abolishing PD-L1 palmitoylation,^196^ thereby improving the efficacy of cancer immunotherapy in a colon tumor model. Due to the interaction of SIGMA I with glycosylated PD-L1, IPAG, the SIGMA I inhibitor restored T cells activity in breast/prostate cancer cells by preventing PD-L1 autophagy.^452^ Furthermore, PD-L1 antibody H1A inhibits PD-L1 interaction with CMTM6, which results in PD-L1 autophagy.^453^

## Conclusions and perspectives

The study of non-apoptotic RCD is an extensive and rapidly developed field. An emerging view is that targeting autophagy, pyroptosis, ferroptosis, and necroptosis in localized tumors profoundly affects the immune cells infiltrated in TME and the response to immunotherapy. Despite the growing importance of ICIs in cancer therapy, their application is greatly limited by the fact that only about one-third of patients respond to ICIs in most cancer types. In order to break the limitations of immunotherapy, in this review, we explore the broad interaction between non-apoptotic cell death mechanisms and antitumor immunity based on the available evidence from laboratory and clinical studies. The roles of autophagy, pyroptosis, ferroptosis, and necroptosis in tumor immunity are still ambiguous as they synergize the antitumor immune response while playing an antagonistic role. In addition, the effects of effectors such as RIPK1/3 and inflammasomes as well as released cytokines and DAMPs through ICD on immune cells and immune response are still controversial. There is a more complex interaction revealed by these findings between non-apoptotic RCD and immunity in different tumor types and contexts. It will also be crucial to understand how distinct TME cell types such as immune cells, tumor cells, and stromal cells interact with each other to inhibit or promote tumor progression by immunity or metabolism reprogramming.

In this context, targeting non-apoptotic cell death seems to be an increasingly promising strategy to improve the efficacy of immunotherapy in the field of cancer therapy. However, it is not certain that non-apoptotic RCD induced by tumoricidal drugs is beneficial for tumor patients in the long perspective because other normal cells could also die when stimulated by DAMPs released from tumor cells through non-apoptotic RCD. Thus, the development of more specific cell death-inducing drugs that act on tumor cells with minimal side effects on normal tissues are extremely urgent. In the meantime, preclinical testing of the order/timing of these drugs in combination with ICIs as well as chemotherapy, radiation, and targeted therapies will be likely crucial to balance therapeutic goals and likely adverse effects. Soon, clinical trials of combination therapies should be actively encouraged to be conducted to assess their efficacy and safety, providing more references for subsequent in-depth studies in order to benefit more cancer patients.
